# International trade shapes global mercury–related health impacts

**DOI:** 10.1093/pnasnexus/pgad128

**Published:** 2023-05-23

**Authors:** Zhencheng Xing, Ruirong Chang, Zhengcheng Song, Yanxu Zhang, Marilena Muntean, Kuishuang Feng, Yifan Liu, Zongwei Ma, Jigan Wang, Jie Zhang, Haikun Wang

**Affiliations:** Joint International Research Laboratory of Atmospheric and Earth System Sciences, School of Atmospheric Sciences, Nanjing University, Nanjing 210023, China; Joint International Research Laboratory of Atmospheric and Earth System Sciences, School of Atmospheric Sciences, Nanjing University, Nanjing 210023, China; Joint International Research Laboratory of Atmospheric and Earth System Sciences, School of Atmospheric Sciences, Nanjing University, Nanjing 210023, China; Joint International Research Laboratory of Atmospheric and Earth System Sciences, School of Atmospheric Sciences, Nanjing University, Nanjing 210023, China; Frontiers Science Center for Critical Earth Material Cycling, Nanjing University, Nanjing 210023, China; Directorate for Energy, Transport and Climate, Air and Climate Unit, European Commission, Joint Research Centre (JRC), Ispra, VA I-21027, Italy; Department of Geographical Sciences, University of Maryland, College Park, MD 20742, USA; State Key Laboratory of Pollution Control and Resource Reuse, School of the Environment, Nanjing University, Nanjing 210023, China; State Key Laboratory of Pollution Control and Resource Reuse, School of the Environment, Nanjing University, Nanjing 210023, China; School of Business, Hohai University, Nanjing 211100, China; School of Business, Hohai University, Nanjing 211100, China; Joint International Research Laboratory of Atmospheric and Earth System Sciences, School of Atmospheric Sciences, Nanjing University, Nanjing 210023, China; Frontiers Science Center for Critical Earth Material Cycling, Nanjing University, Nanjing 210023, China; Collaborative Innovation Center of Climate Change, Jiangsu Province, Nanjing 210023, China

**Keywords:** mercury, international trade, atmospheric transport, health impact

## Abstract

Mercury (Hg) is a strong neurotoxin with substantial dangers to human health. Hg undergoes active global cycles, and the emission sources there of can also be geographically relocated through economic trade. Through investigation of a longer chain of the global biogeochemical Hg cycle from economic production to human health, international cooperation on Hg control strategies in Minamata Convention can be facilitated. In the present study, four global models are combined to investigate the effect of international trade on the relocation of Hg emissions, pollution, exposure, and related human health impacts across the world. The results show that 47% of global Hg emissions are related to commodities consumed outside of the countries where the emissions are produced, which has largely influenced the environmental Hg levels and human exposure thereto across the world. Consequently, international trade is found to enable the whole world to avoid 5.7 × 10^5^ points for intelligence quotient (IQ) decline and 1,197 deaths from fatal heart attacks, saving a total of $12.5 billion (2020 USD) in economic loss. Regionally, international trade exacerbates Hg challenges in less developed countries, while resulting in an alleviation in developed countries. The change in economic loss therefore varies from the United States (−$4.0 billion) and Japan (−$2.4 billion) to China (+$2.7 billion). The present results reveal that international trade is a critical factor but might be largely overlooked in global Hg pollution mitigation.

**Significance Statement**

Effective international mercury mitigation requires a good knowledge of the longer global biogeochemical mercury cycle from economic activities to human health. Here, we combine four global models to investigate the effect of international trade on the relocation of mercury pollution and related human health impacts across the world. We find that international trade enables the whole world to avoid considerable health impacts and economic loss, but this comes at the cost of environmental degradation in less developed countries. Our results highlight the role of international trade in international cooperative mercury control.

## Introduction

Mercury (Hg) is a potent neurotoxic substance, since the organic form thereof, methylmercury (MeHg), can result in neurocognitive abnormalities in kids and cardiovascular issues in adults (1, 2). In the annals of history, MeHg exposure has resulted in substantial health concerns to human beings. For example, 1,784 people died of the Minamata disease in Japan (3). More than 10,000 deaths from fatal heart attacks (FHA) are thought to occur each year in China as a result of exposure to MeHg (4). It is calculated that MeHg exposure costs the United States and Europe $16 billion in lost productivity due to the intelligence quotient (IQ) decline of developing brains (5, 6). About 60% of the Hg emitted to the atmosphere each year derives from anthropogenic Hg that was previously deposited in soil and water (7). Just 10% of the current emissions come from natural sources, and the remaining 30% come from manmade sources (8). Human-related Hg emissions play a sizable part in the global Hg budget (9). Among the background of such context, the Minamata Convention on Mercury was signed by 128 countries to establish a mandate for measures to combat global Hg contamination (10), including reducing artisanal and small-scale gold mining (ASGM) as well as the purposeful use of Hg in goods and industrial processes.

Through international trade, locations where commodities are consumed are separated from those where emissions and the resulting pollution and health effects occur (11, 12). Although items from downstream industries don't contain Hg, using these products can cause upstream Hg emissions from the manufacture of raw and intermediate materials (13, 14). Moreover, developed economies frequently transfer Hg emissions to emerging countries in order to boost financial returns by utilizing cheap labor and energy as well as lax environmental regulations, which can be attributed to differences in division of labor in the global value chains (15, 16). As such, there is debate among academic circles concerning whether existing production-side controls (for example, putting up Hg removal facilities and adopting cleaner production techniques) can effectively reduce emissions, since the drivers (that is, consumption) behind such emissions are neglected (17, 18). Thus, the role of international trade should be identified in the relocation of global Hg emissions, pollution, exposure, and related health burden.

A series of processes, such as Hg emission, atmospheric transport and deposition, environmental exchange, Hg methylation, food web transfers, and dietary intake, affect MeHg exposure to humans (19). The various phases of the Hg cycle are interrelated and may have an impact on one another. Several specific segments of the chain have been evaluated in previous studies. For example, Hg emissions are generated from production activities and induced by consumers through international trade (20, 21). The physical transport of Hg emissions from other countries can significantly affect domestic Hg pollution (22, 23). Hg levels in environmental medias can influence the MeHg levels in various foods including seafood, freshwater fish, and rice (24, 25). MeHg intake in one place may result from a variety of meals from distinct worldwide regions (26–28). Despite the aforementioned findings, the global biogeochemical Hg cycle has not been identified along a longer path from economic production to human health.

Notably, the effects of subnational trade on Hg emissions, transportation, deposition, food exposure, and related health impacts have been investigated in China (4). However, in such research, the atmospheric Hg deposition was taken as the sole proxy to scale the change of food MeHg levels without inclusion of marine plankton and soil Hg levels which are more suitable proxy indicators for seafood and rice (29). At the same time, studies focusing on a sole country result in a failure to provide effective policy instruction for the implementation of the Minamata Convention from a multinational perspective. The limitations in previous studies make it difficult to identify how international trade affects the Hg-related health risks and to propose practical policy solutions to mitigate those risks through cooperation on a global scale. In this context, a comprehensive investigation of the Hg health impacts associated with global trade is urgently needed for promoting international cooperation on the Minamata Convention (30).

In the present study, the extent to which international trade results in relocation of Hg emissions, pollution, exposure, and related health impacts across the world is evaluated, and the net effect thereof on the global Hg challenges is further investigated. Such efforts are achieved by linking a Hg emission inventory (31), a global multiregional input–output (MRIO) model (32, 33), a coupled atmosphere–land–ocean–ecosystem model (29), and an exposure–risk–valuation model (34). Notably, it is assumed that a certain country's MeHg exposure via seafood is proportional to the global marine plankton MeHg levels weighted by its spatial distribution of fish catch rather than a simple global average concentration adopted by Zhang et al. (29). This enhancement may help improve the accuracy of food MeHg exposure estimates for various countries. The present method allows for a more comprehensive assessment and could be used to look into a longer chain of the global biogeochemical Hg cycle from economic production to human health. The results may facilitate the implementation of an ecological compensation mechanism to advance international cooperation on Hg control strategies in the Minamata Convention and formulate targeted emission reduction measures for different countries from both production and consumption perspectives (further details are provided in the policy implications in a later section).

## Results and discussion

### Relocation of Hg emissions of countries

**Fig. 1.**
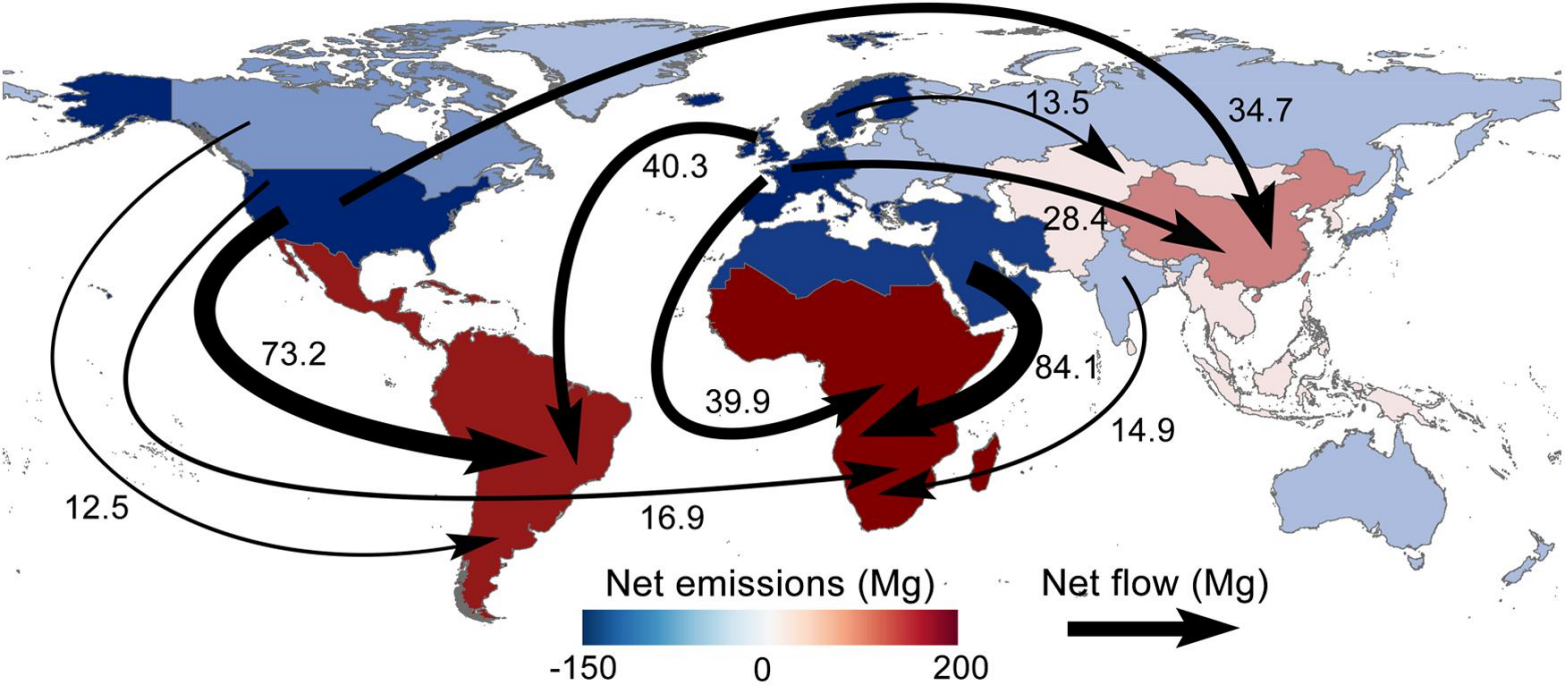
Spatial distributions of net Hg emissions embodied in the international trade and prominent net flows of Hg emissions between the 13 world regions. Bilateral flows of Hg emissions embodied in the international trade between 13 world regions are plotted in Fig. S3. The shading of regions represents the difference between where Hg emissions are physically produced and where the related goods and services are consumed, thereby highlighting the emissions embodied in the net international trade. Arrows show the 10 largest net flows of embodied Hg emissions, and the sizes of arrows correspond to the amount of net emission flows.

Globally, human activities release a total of 1,833.3 Mg of Hg in 2011 (31), 47.1% (864.2 Mg) of which is embodied in international trade. In other words, nearly half of the global Hg emissions can be linked to exports to other countries (see Fig. S1 for emissions embodied in bilateral trade). Figure 1 displays the net impact of international trade on Hg emissions in 13 world regions (see Fig. S2 for regional definitions). The top net Hg receptors are all less developed economies (red shading, Fig. 1), such as sub-Saharan Africa (+199.4 Mg), Latin America (+166.3 Mg), and China (+80.9 Mg). Such economies are found to be major sources of global Hg emissions (Fig. S3), of which China is the world's largest emitter (546.1 Mg), followed by sub-Saharan Africa (291.8 Mg) and Latin America (291.3 Mg). From the view of sector, the smelting and pressing of nonferrous metals are the primary direct contributors to the Hg emissions of the three regions, even accounting for over 90% in sub-Saharan Africa (Fig. S3). The aforementioned findings can primarily be attributed to the heavy use of Hg by the ASGM in such regions (35). The top net Hg outsourcers are mainly developed economies (blue shading, Fig. 1), such as the United States (−145.2 Mg), Western Europe (−140.9 Mg), and Japan (−36.9 Mg). However, several developing economies such as the Middle East and North Africa (−112.7 Mg), India (−20.6 Mg), and Eastern Europe (−5.9 Mg) are also found with net negative emissions, which could be ascribed to their lower emissions thereof from ASGM, thereby contributing to reducing production-based emissions (Fig. S3).

Developing economies, the main suppliers of primary and semimanufactured products (for example, metals and nonmetallic mineral products), are located upstream of the global supply chains. In contrast, industrialized economies have higher consumption-based emissions since they are further away from global supply chains (Fig. S3). The specialization of global value chains results in a vast difference between the Hg emission intensities of industrialized and emerging economies (Fig. S4). As such, flows of Hg emissions induced by international trade are found to mainly go from developed to developing economies (Fig. 1). For example, Middle East and North Africa outsources 84.1 Mg of net emissions to sub-Saharan Africa, followed by the United States → Latin America (73.2 Mg), Western Europe → Latin America (40.3 Mg), Western Europe → sub-Saharan Africa (39.9 Mg), and the United States → China (34.7 Mg). These results demonstrate that developed economies can avoid significant amounts of Hg emissions by transferring Hg-intensive production activities to emerging economies through international trade (18), thereby highlighting a necessity for international responsibility sharing on Hg control from an environmental equity perspective.

### Redistribution of environmental Hg levels

**Fig. 2.**
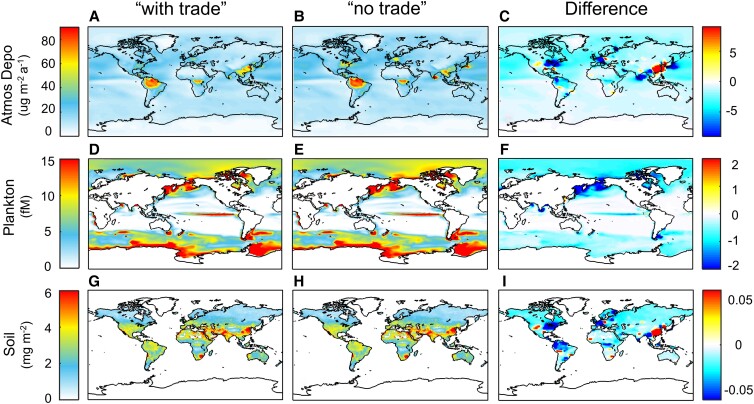
Global environmental Hg levels under “with trade” and “no trade” scenarios and the differences there between. The differences between global environmental Hg levels due to the emissions produced in each region and those due to the emissions related to the consumption in each region denote the net impact of international trade on environmental Hg levels. The rows (A–C), (D–F), and (G–I) are for atmospheric deposition, marine plankton, and soil concentration, respectively. For each row, the three panels denote the spatial distribution of environmental Hg levels for “with trade” and “no trade” scenarios and the differences therebetween. The color bars on the right-hand side share the same units with the corresponding color bars on the left-hand side.

One central question is whether the international trade has inadvertently increased or decreased global Hg pollution relative to a world with no trade. In the present study, to assess the effects of international trade on global Hg pollution, a counterfactual scenario with the absence of international trade (“no trade” scenario) is set up for comparison with the existence of international trade (“with trade” scenario). There is an assumption that trade partners can manufacture the same commodities that are originally imported through international trade at the same emission level (4, 12). As such, the global total emissions remain unchanged, but emissions of various countries are counted according to consumption rather than production. See Table S1 for more detailed descriptions. The gridded emission inventory under the “with trade” scenario and production- and consumption-based emissions of countries are combined to scale the corresponding gridded emissions for each country under the “no trade” scenario. The Hg emission inventory under “with trade” and “no trade” scenarios and the differences therebetween are provided in Fig. S5. Once released into the environment, Hg can diffuse and move through various environmental medias (for example, the atmosphere, water, and soil). Figure 2 shows the environmental Hg levels under “with trade” and “no trade” scenarios and the differences therebetween, indicating the net impact of international trade on the distribution of Hg pollution. Findings are made that the relocation of emissions induced by international trade has, to varying degrees, redistributed atmospheric Hg deposition, soil Hg concentrations, and marine planktonic MeHg over the world.

International trade is found to considerably redistribute atmospheric Hg deposition globally, with annual deposition flux ranging from −40.8 Mg (United States) to +44.9 Mg (China). This results in the transfer of atmospheric Hg pollution from developed countries to less developed ones (Fig. 2A–C). Despite being similar to the relocation of emissions (Fig. 1), the relocation of atmospheric Hg deposition also highlights the case for which there are neighboring areas downwind of where the emissions are generated. For instance, emissions embodied in Chinese exports contribute to downwind Hg deposition over the Northwest Pacific Ocean and East Asian countries [for example, South Korea and Japan; Chen et al. (4)]. Overall, the countries with reduced Hg deposition (for example, the United States, Japan, and Western Europe) have a larger decrease in deposition than other countries with increased deposition (for example, China and Latin America), thereby resulting in a net decline in global total Hg deposition (−244.1 Mg). Indeed, atmospheric deposition occurs in several pathways including dry deposition of elemental (Hg^0^) and oxidized Hg (Hg^II^) and wet deposition of Hg^II^, which are mainly influenced by the atmospheric redox chemistry, land use/cover characteristics, and meteorological factors. In general, atmospheric Hg^II^ is more readily deposited with a greater local impact than Hg^0^ (7, 9). Additionally, the oxidation rate of atmospheric Hg is found to be higher in areas around 40°N than those around 0° (Fig. S6), mainly due to the difference in the levels of regional oxidizing substances (36). As such, the trade-induced transfer of emissions from high-latitude (40°N) to low-latitude areas (0°), such as Middle East and North Africa → sub-Saharan Africa (84.1 Mg) and the United States → Latin America (73.2 Mg), as shown in Fig. 1, results in a decline in the oxidation degree of global atmospheric Hg. Consequently, international trade leads to an overall decline in global total Hg deposition, even though the two scenarios have the same global total Hg emissions.

Marine planktonic MeHg is also significantly sensitive to international trade (Fig. 2D–F). Moreover, the percentage variation of planktonic MeHg levels is comparable with Hg deposition, because the substrate of MeHg in the ocean (i.e. inorganic Hg) originates primarily from atmospheric deposition (Fig. 2F; 37). For instance, the trade-induced increase in spillover from Hg deposition of China contributes to the increase in planktonic MeHg concentrations over the seas leaving the eastern terrestrial boundary, including the Chinese coastal seas and the Sea of Japan (Fig. 2F). Contrarily, the trade-induced decrease in atmospheric deposition of developed countries (for example, Japan and Western Europe) leads to the decrease in planktonic MeHg concentrations in the corresponding sea areas thereof, such as the Sea of Okhotsk and the Arctic Ocean. Globally, the average plankton MeHg concentrations for the “with trade” scenario are also lower than the “no trade” scenario (Fig. S7), which could be attributed to the aforementioned atmospheric deposition changes. The variations in soil Hg concentrations are substantially lower (<1%, Fig. 2I) due to the enormous mass and prolonged lifetime of Hg in the reservoir (38, 39). Nevertheless, owing to the lower atmospheric deposition, the global soil Hg levels are also found to be lower under the “with trade” scenario (Fig. S7).

### Relocation of Hg exposure and related impacts

**Fig. 3.**
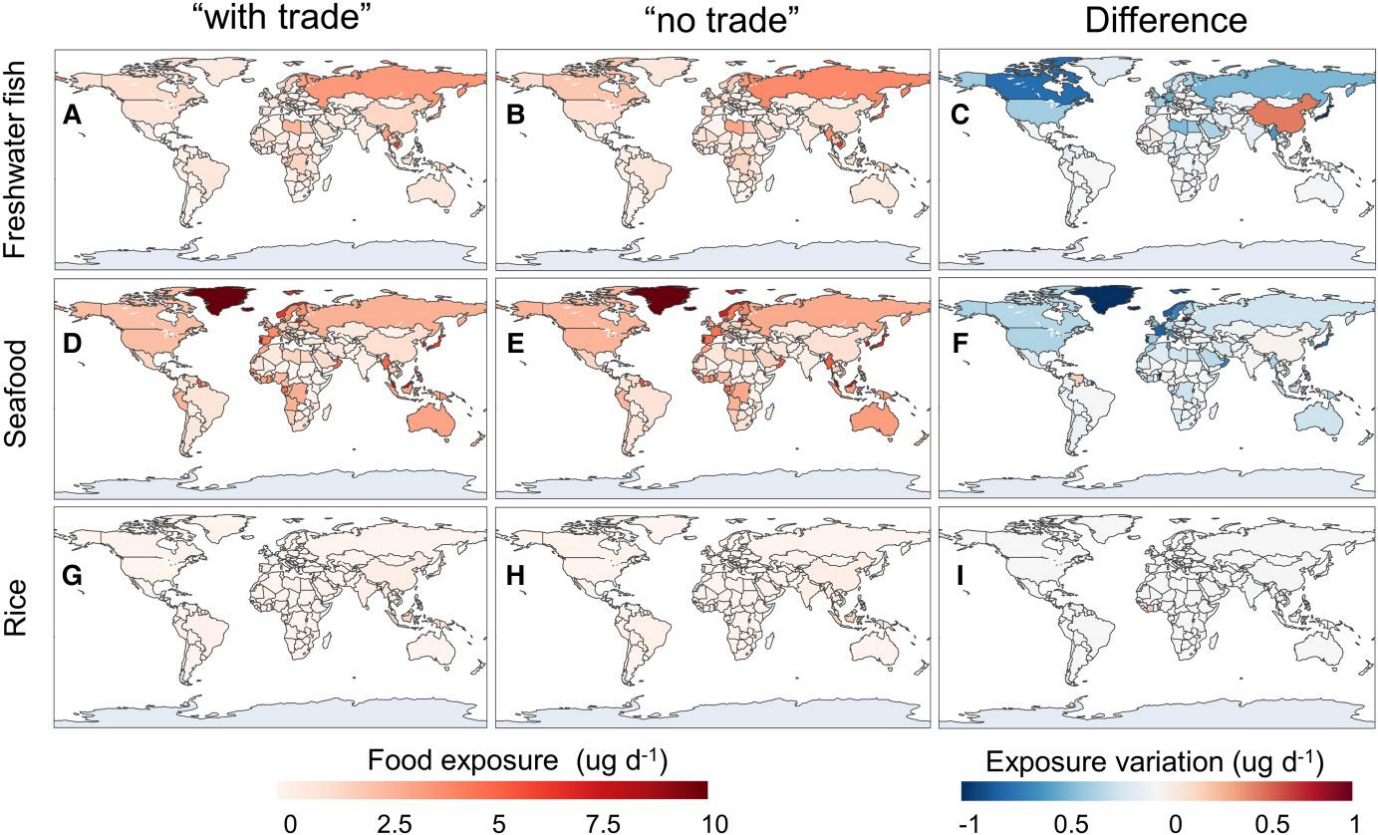
Food MeHg exposure of countries under “with trade” and “no trade” scenarios and the differences therebetween. The rows (A–C), (D–F), and (G–I) are for freshwater fish, seafood, and rice MeHg exposure, respectively. For each row, the three panels denote the regional distribution of food exposure for “with trade” and “no trade” scenarios and the differences therebetween.

The MeHg exposure levels from food consumption of three categories including seafood, freshwater fish, and rice are included in the present study along with the MeHg concentrations from previous research (29). The exposure levels under the “with trade” scenario and the modeled environmental Hg levels are combined to scale the MeHg exposure under the “no trade” scenario (see Materials and methods). Figure 3 shows the food MeHg exposure levels under “with trade” and “no trade” scenarios and the differences therebetween indicating the net impact of international trade on the exposure of countries. As the largest source of human MeHg exposure, seafood intake accounts for 56% of the global total food exposure, followed by freshwater fish (35%) and rice (9%), which is mainly attributed to the differences in Hg bioaccumulation between the three food categories (28). At the same time, as atmospheric Hg deposition and marine planktonic MeHg are both significantly sensitive to international trade (Fig. 2), changes in exposure from seafood and freshwater fish are even more substantial (Fig. 3). As an example, the trade-induced relocation of deposition leads to an increase of 0.51 *µ*g/d in freshwater fish exposure for China and a decline of 1.66 *µ*g/d in Japan (Fig. 3C). The decline in marine planktonic MeHg enables coastal countries that consume a lot of seafood to achieve considerable decreases in seafood MeHg exposure (Fig. 3F), such as the Maldives (−2.40 *µ*g/d), Iceland (−1.40 *µ*g/d), Haiti (−1.36 *µ*g/d), and Qatar (−1.31 *µ*g/d). The change in MeHg exposure via rice is less obvious for all the countries (Fig. 3I), which could be attributed to the limited changes in soil Hg concentrations (Fig. 2I). Overall, as shown in Fig. S8, the trade-induced redistribution of environmental Hg levels leads to a net decline in global total exposure (−0.14 *µ*g/d), with seafood (−0.10 *µ*g/d) and freshwater fish (−0.04 *µ*g/d) being the main contributors.

**Fig. 4.**
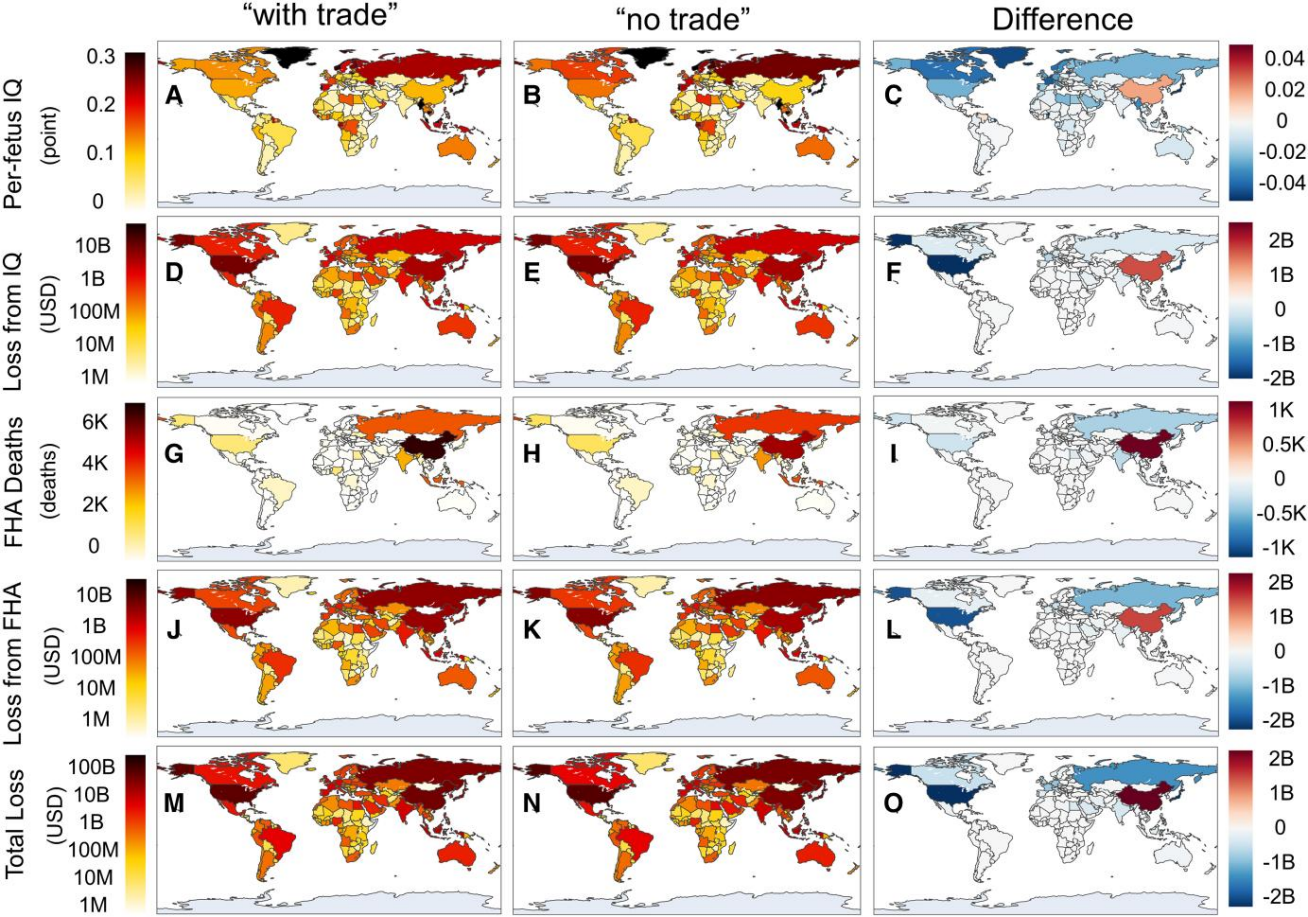
MeHg exposure-related health and economic impacts of countries under “with trade” and “no trade” scenarios and the differences therebetween. (A–C) Per-fetus IQ decrement; (D–F) economic loss from IQ decrement; (G–I) FHA deaths; (J–L) VSL loss from FHA; and (M–O) total loss from MeHg exposure. Economic losses are in US dollars (2020 value and adjusted by purchasing power parity). The gray area indicates missing data. The color bars on the right-hand side share the same units with the corresponding color bars on the left-hand side.

Two health outcomes are included as a result of food MeHg exposure: decline in infant IQ and FHA mortality. These endpoints are estimated according to the total exposure of the aforementioned pathways from seafood, freshwater fish, and rice. Based on the population and economic growth data of individual countries, the IQ decline is converted to a loss in lifetime wages. The economic loss from FHA is calculated by using a value of the statistical life (VSL) approach, which is derived from the GDP per capita of various countries (see Materials and methods). Figure 4 shows MeHg exposure-related human health and economic impacts of countries under the “with trade” and “no trade” scenarios and the differences therebetween, indicating the net impact of international trade. As shown in Fig. 4C, the largest increase in IQ decrement per fetus is in the Bahamas (+0.036 points), Latvia (+0.028 points), and China (+0.020 points), while the largest decrease occurs in Japan (−0.087 points), the Maldives (−0.086 points), and Qatar (−0.086 points), which is mainly related to the change in food MeHg exposure (Fig. 3). Taking into consideration the birth rates and income levels of various countries, the change in economic loss attributed to IQ decline of newborns could be obtained (Fig. 4F). China is found with the largest increase in economic loss (+$1.3 billion) as a result of trade, while the largest decrease occurs in the United States (−$2.3 billion) and Japan (−$1.6 billion).

The regional pattern of the mortality related to FHA (Fig. 4I) differs significantly from that of IQ decline per fetus, with the inclusion of total population and baseline FHA incidence. The largest changes in deaths attributed to trade are from populous countries with significant variation in the exposure, such as China (+979 deaths), Russia (−322 deaths), India (−253 deaths), the United States (−204 deaths), and Japan (−130 deaths). Considering the difference in VSL per death, the United States has the highest loss reduction from such pathway (−$1.7 billion), followed by Russia (−$0.92 billion), Japan (−$0.81 billion), and Germany (−$0.43 billion), while China is faced with an increase of $1.4 billion in economic loss (Fig. 4L). Combining the two endpoint’s results, China suffers from an increase of $2.7 billion per year in total loss, while developed countries obtain considerable alleviation in total loss, such as the United States (−$4.0 billion), Japan (−$2.4 billion), and Russia (−$1.2 billion), as shown in Fig. 4O.

### Decline in global Hg-related health risks

We further calculate global changes across all countries and assess the net effects of the international trade. As shown in Table 1, international trade enables the whole world to avoid 5.7 × 10^5^ points of IQ decrements (0.41 × 10^−2^ points per fetus) and 1,197 deaths per year from FHA. Taken together, global economic loss associated with MeHg exposure decreased by $12.5 billion (2020 USD). Given the strong assumptions on the scenario setting, these results should be interpreted cautiously, but they, to a certain extent, examine the effects of international trade on mitigating global Hg pollution and related health impacts.

**Table 1.** Global Hg-related human health and economic impacts under actual “with trade” and counterfactual “no trade” scenarios and the differences therebetween.

	Actual “with trade” scenario	Counterfactual “no trade” scenario	Net impacts of international trade
Per-fetus IQ decrements (point)	8.56 × 10^−2^	8.97 × 10^−2^	−0.41 × 10^−2^
IQ decrements (point)	1.17 × 10^7^	1.23 × 10^7^	−5.66 × 10^5^
FHA deaths (deaths)	28,968	30,165	−1197
Economic loss from IQ (billion USD)	61.0	68.1	−7.1
VSL loss from FHA (billion USD)	56.9	62.4	−5.5
Total loss (billion USD)	118.0	130.0	−12.5

**Fig. 5.**
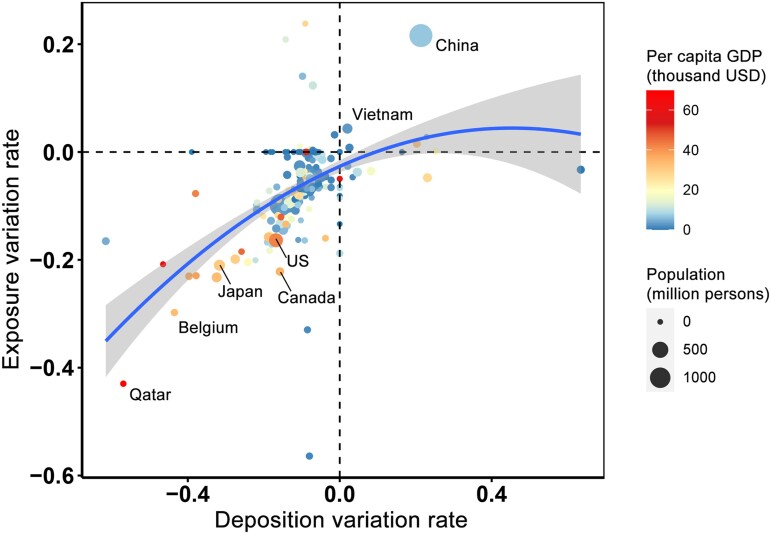
Contrasting changes in atmospheric Hg deposition and MeHg exposure across the world. The figure shows the relative changes in regional Hg deposition and MeHg exposure resulting from international trade for each country. The color of the dots denotes the magnitude of per capita GDP, and the size of the dots denotes the amount of population.

As aforementioned, international trade is found to considerably relocate the distribution of Hg emissions, pollution, MeHg exposure, and related health risks among various countries, without changing the total emissions of the world. However, the trade-induced relocation of emissions results in a nonnegligible reduction in global gross Hg deposition (−244.1 Mg, −8.2%), which, to a certain extent, contributes to the alleviation in global total food MeHg exposure and related health impacts. Additionally, a regional asymmetry of variations in MeHg levels in food products and subsequent MeHg exposure over various deposited regions is also brought on by shifts in the spatial distributions of the deposition. There are significant differences in landscape patterns and dietary structure between countries which may influence the extent of the deposition of Hg to the sea and the intake of MeHg to the body, respectively. When compared with inland rice-growing areas (for example, sub-Saharan Africa), coastal fish-eating areas (for example, Western Europe) may suffer a higher impact from the same quantity of atmospheric Hg deposition. Significant differences exist in the sensitivity between Hg deposition and MeHg exposure variations across the world, wherein developed countries are found to be more risk prone than less developed countries (Fig. 5). As the exposure and health burden variations are assumed to present a linear relationship (Fig. S9), the trade-driven transfer of Hg from coastal developed countries to inland developing countries can lead to a reduction in global Hg-related health risks. Notably, despite being a developing country, China is found to be relatively risk prone (Fig. 5). Such findings could be attributed to the trade-induced increase in the Hg deposition thereof contributing to the increase in planktonic MeHg concentrations over the seas leaving the eastern terrestrial boundary where there is a dense population whose diets contain many aquatic food products (4). As a result, 99% of the increased exposure in China is attributable to the consumption of seafood and fish products (Fig. S10).

### Uncertainties and limitations

The compilation of Hg emission inventory, the estimation of emissions embodied in trade, the simulation of the Hg transport model, the gathering of data on food consumption and MeHg concentrations, and the assessment of the effects on human health and monetary loss are the main sources of uncertainty that can affect the results of our model. Muntean et al. (40) reported that global production-based anthropogenic emissions have an overall uncertainty of [−33%, 42%] which serves as an important reference for this study. A recent study reported uncertainty (coefficient of variation) of Chinese provincial Hg footprint varying from 8 to 34% by using the Monte Carlo simulation method (15). As the uncertainty for small emitters is larger than that of large emitters (41), the uncertainty for national Hg footprint in our study should be lower than 34%. Based on the simulated and observed results of environmental Hg levels (Fig. S11), the normalized root–mean–square deviation (NRMSD) is applied to calculate the uncertainty caused by the Hg transport model (see Supplementary material). For example, the uncertainty for surface total gaseous Hg is estimated at 29.1%.

Referring to our previous work (29), the uncertainty of food consumption is estimated at [−47%, 42%], by comparing the FAO data with national data. The uncertainty of food MeHg concentration is [−37%, 63%], by considering its log-normal distributions. The dose–response relationships between MeHg exposure and health consequences make up the largest portion of the uncertainty, with a range in [−59%, 147%]. An uncertainty of [−70%, 26%] for economic loss results from the factors for economic value, such as the VSL of FHA deaths. We take into account how the dose–effect relationship, economic valuation, food consumption, and MeHg concentrations contribute to overall uncertainty. A Monte Carlo method is used to estimate the overall degree of uncertainty. The settings for these four components are randomly picked, and the health risk computation is repeated 1,000 times. The computed risk's 2.5 and 97.5% percentiles are used to determine the overall uncertainty range (or 95% confidence interval). The overall uncertainty for total economic loss is estimated at [−75.3%, 131.6%].

Although the uncertainty of estimates under the “with trade” and “no trade” scenarios is relatively high, the effect of international trade is expected to have a lower uncertainty, as the derivation of the difference between the estimates under the two scenarios may offset some of their original uncertainty. Moreover, despite the uncertainties from various stages, our comprehensive models, to a certain extent, advance the knowledge of the global biogeochemical Hg cycle from economic production to human health, and the results are more scientific and reliable compared with some more simplified models. In the future research, setting a more realistic “no trade” scenario considering the distinctions of production structures, ecological endowment, and commodity prices among countries can help reduce the uncertainty. Additionally, investigation on the Hg pollution and health impacts embodied in both international and intranational trades of subcountry regions (e.g. Chinese provinces) is meaningful for the implementation of emission reduction measures. The adoption of nested national–global MRIO model can help produce some more instructive and practical policy measures for a certain country.

### Policy implications

A map of the global health risks brought on by trade-induced Hg emissions is identified from the more comprehensive biogeochemical Hg cycle at the global scale. International trade considerably relocates the regional distribution of Hg challenges across the world, which enables the whole world to avoid considerable Hg-related health risks, while also resulting in contrary changes in health loss between developed and developing economies. To expand further, some of the gains from Hg emission reduction in developed economies are likely to be at the expense of the ecosystem and human health in developing economies, which is a critical inequity issue between developed and developing countries. However, the transfer of heavy industries (for example, smelting and pressing of metals) from sensitive areas (for example, the coast of developed countries) to less sensitive areas (for example, inland regions of less developed countries) could alleviate the global total MeHg exposure. As such, a fair and effective responsibility sharing and compensation mechanism need to be established for trade-induced Hg-related health risks, so as to advance international cooperation on the implementation of the Minamata Convention on Mercury. For instance, organizations such as the United Nations and the World Health Organization could establish an international compensation fund in which countries benefiting from reduction in Hg emissions should compensate the countries suffering losses based on changes in economic loss resulting from international trade.

The understanding of Hg-related dangers embedded in global trade expands upon the present mitigation strategies for Hg, which solely aim to reduce direct emissions, and thus offers opportunities for reducing indirect emissions. To mitigate Hg-related health risks in an effective way, targeted policy methods should be implemented at various stages of global supply chains. Production-side controls work well in key regions and sectors with huge direct Hg emissions and significant health concerns. These controls include increasing energy efficiency, phasing out outdated factories, and building Hg removal facilities. As an example, these steps are successful for sources like nonmetal mineral products, smelting and pressing of metals, and production and supply of electricity and heat power in sub-Saharan Africa, Latin America, and China. Demand-side control strategies work well in key regions and sectors whose final consumption is responsible for significant upstream health hazards. These strategies include adjusting consumption taxes to influence consumer behavior. For example, demand-side actions are more frequently thought about when products from the construction, general and special equipment, and electric equipment and machinery sectors are consumed. Regionally, developed economies, such as the United States, Western Europe, Middle East, and North Africa, should prioritize implementation of these demand-side control measures.

## Materials and methods

### Framework of the global Hg risk assessment model

**Fig. 6.**
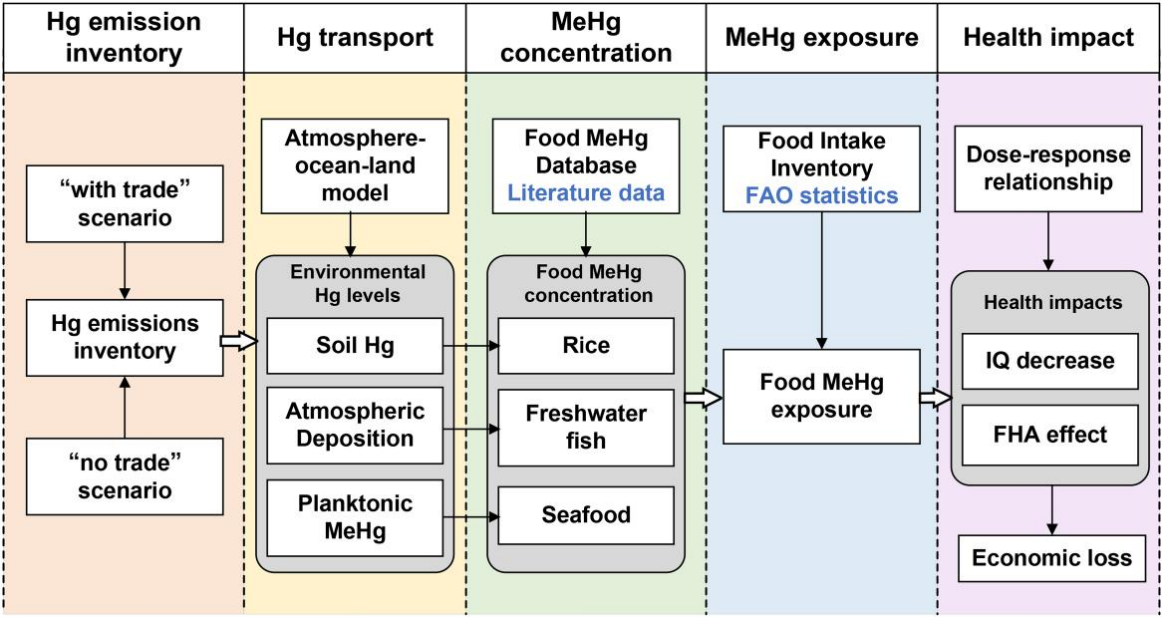
Methodology framework to access Hg-related impacts.

A more comprehensive evaluation methodology is established in this work to characterize the biogeochemical Hg cycle from economic production to human health. As such, we identify the role of international trade in relocation of global Hg emissions, pollution, exposure, and related health burden. As illustrated in Fig. 6, the model comprises five components: Hg emission inventory, Hg transport model, food MeHg concentrations, MeHg exposure, and human health impacts. We provide detailed descriptions of the methods used for each component in the following sections.

### Hg emission inventory

To track the emissions embodied in globally traded commodities, a thorough sectoral disaggregation of production-based emissions is required. One crucial aspect of this process is accounting for atmospheric Hg emissions, which are mainly the result of human production activities. These activities include burning fuel, smelting nonferrous metals, making building materials, mining for primary Hg, and burning waste. Hg emission inventory in EDGARv4.tox2 provides Hg emissions from 14 sectors (see Table S2) of 225 countries. In order to match these primary anthropogenic sources to MRIO sectors in GTAP, we aggregate the 65 sectors in GTAP into 39 aggregated sectors (see Table S3). For example, three sectors from GTAP (i.e. manufacture of chemicals and chemical products, manufacture of pharmaceuticals, medicinal chemical and botanical products, and manufacture of rubber and plastics products) are aggregated into the aggregated sector, manufacture of chemicals and chemical products. Then, Hg emissions from the source of production of chemicals in EDGAR are counted to the sector. By analogy to the method, the other sources of Hg emissions in EDGAR are counted to their corresponding sectors (see Table S4). As such, we obtain production-based Hg emissions by country and by sector, which can be combined with MRIO to calculate the consumption-based emissions of countries.

Then, the MRIO method is employed to estimate consumption-based Hg emissions. MRIO has been extensively used in studying environmental consequences embedded in trade (42, 43) as it can depict the flows of commodities along the supply chain between various industries and economies (44, 45). The Hg emissions flowing from importing regions (i.e. final consumers) to exporting regions (i.e. direct emitters) through global supply chains can be computed by Eq. (1). Detailed derivations of the formula refer to previous studies (46).


(1) $$EE_{Y^{s}}^{r}=E^{r}L^{r}Y^{s}(r\neq s),$$

where $E^{r}$ denotes the row vector of Hg emission coefficients (defined as the ratio of emissions produced to economic output in each sector) of the region *r*; $L^{r}$ is the submatrix in L for the region; $L=(I-A{)}^{-1}$ is the Leontief inverse matrix; A stands for the direct consumption matrix, and each column of A denotes the amount of input needed from each sector to produce one unit output of this column sector; $Y^{s}$ denotes the final consumption vector of region *s*.

### Hg transport model

The fate and transport of Hg across various environmental compartments, including the climate, atmosphere, land, ocean, and marine ecosystem, are simulated using a linked Earth system model framework (29). The framework consists of three models: a 3D atmospheric model (GEOS-Chem), a 3D ocean model (MITgcm), and a 2D terrestrial model (GTMM). The GEOS-Chem model is used to simulate atmospheric transport, as well as its dry and wet deposition. Additionally, the model also accounts for the redox chemistry of Hg which includes three forms of Hg (i.e. gaseous elemental, gaseous oxidized, and particle-bound Hg). The model is based on version 13.3.0 featuring a horizontal resolution of 4° × 5° and 47 vertical layers (36). The fate of Hg in the soil pool and the land–atmosphere exchange is simulated by GTMM (39) which covers a single layer of the top 30 cm with a resolution of 1° × 1°. The transport, chemistry, and trophic transfer of Hg in the ocean are simulated by the MITgcm (37), which covers 50 vertical levels with a horizontal resolution of ∼1° × 1°. The NJUCPL is used to connect the three models online and exchange Hg among environmental media on an hourly time step (47). GEOS-Chem provides atmospheric Hg concentration and deposition data to GTMM and MITgcm, while GTMM and MITgcm separately pass soil reemission and ocean evasion fluxes to GEOS-Chem. Detailed descriptions of these models refer to our previous work (29) and related references therein. We run the coupled model for a 10-year period with the Hg emission inventory under “with trade” and “no trade” scenarios in 2011 based on the meteorological condition in the period between 2004 and 2014.

### Food MeHg exposure

The actual MeHg exposure under the “with trade” scenario for human beings in each country is derived from our previous studies (29). This study takes into account MeHg exposure from seafood, freshwater fish, and rice. We gather the information for the typical MeHg levels in these foods from the literature (29). The FAO database is used to collect the food intake inventory for each nation. To calculate the country-specific total MeHg exposure, we add up the product of MeHg levels and food intake for each food category by using Eq. (2).


(2) $$E_{\mathrm{WT}}=I_{\mathrm{seafood}}C_{\mathrm{seafood}}+I_{\mathrm{fwfish}}C_{\mathrm{fwfish}}+I_{\mathrm{rice}}C_{\mathrm{rice}},$$

where $E_{\mathrm{WT}}$ denotes the actual MeHg exposure under the “with trade” scenario; $I_{\mathrm{seafood}}$, $I_{\mathrm{fwfish}}$, and $I_{\mathrm{rice}}$ denote the food intake for seafood, freshwater fish, and rice, respectively; $C_{\mathrm{seafood}}$, $C_{\mathrm{fwfish}}$, and $C_{\mathrm{rice}}$ are the MeHg levels for seafood, freshwater fish, and rice, respectively.

It has been demonstrated that MeHg concentrations in ecosystems are determined by Hg levels in environmental media in the previous studies (29). We use the literature-obtained food MeHg concentrations under “with trade” scenario and the modeled environmental Hg levels to scale the MeHg levels under “no trade” scenario. The freshwater fish MeHg concentration for a certain country is assumed to be proportional to its population-weighted Hg deposition (D; 48). The rice MeHg concentration is assumed to be proportional to the total soil Hg concentration (S; 49). For the seafood, the MeHg exposure of a certain country is assumed to be proportional to the global ocean plankton MeHg levels weighted by its spatial distribution of fish catch (P; 50, 51). Assuming that the food consumption patterns remain the same, the MeHg exposure under “no trade” can be computed as follows:


(3) $$E_{\mathrm{NT}}=\frac{P_{\mathrm{NT}}}{P_{\mathrm{WT}}}I_{\mathrm{seafood}}C_{\mathrm{seafood}}+\frac{D_{\mathrm{NT}}}{D_{\mathrm{WT}}}I_{\mathrm{fwfish}}C_{\mathrm{fwfish}}+\frac{S_{\mathrm{NT}}}{S_{\mathrm{WT}}}I_{\mathrm{rice}}C_{\mathrm{rice}},$$

where $E_{\mathrm{NT}}$ denotes the hypothetical MeHg exposure under the “no trade” scenario; $P_{\mathrm{WT}}$, $D_{\mathrm{WT}}$, and $S_{\mathrm{WT}}$ denote the plankton MeHg concentration in the ocean, Hg atmospheric deposition, and soil Hg concentration under the “with trade” scenario, respectively; $P_{\mathrm{NT}}$, $D_{\mathrm{NT}}$, and $S_{\mathrm{NT}}$ denote the plankton MeHg concentration in the ocean, Hg atmospheric deposition, and soil Hg concentration under the “no trade” scenario, respectively.

### Human health impact

In this study, two health consequences of MeHg exposure are taken into account: decline in neonatal IQ and FHA. When assessing the potential impact of MeHg exposure on IQ, a linear dose–response relationship is used, which assumes that any level of exposure can lead to a corresponding decrease in IQ without a safe threshold level (34):


(4) ΔIQ=γλβ×ΔE×BW,

where ΔIQ is the decline in IQ points, ΔE denotes the variation in dietary food MeHg intake, and BW represents the body weight. The coefficient *β* links food intake to blood Hg levels, which is further linked by *λ* to the Hg levels in hair. The coefficient *γ* denotes the dose–response association between hair Hg levels and IQ decrement.

For the FHA effect, a log-linear relationship is adopted:


(5) $$\Delta \mathrm{CF}=\sum_{g}\mathrm{PO}P_{g}\times Cf_{g}\times \omega \times (1-e^{-\varphi \lambda \beta \times \Delta E\times \mathrm{BW}}),$$

where ΔCF denotes the changes in the FHA deaths linked to MeHg exposure; $\mathrm{PO}P_{g}$ and $Cf_{g}$ represent the population and baseline FHA incidence rate of gender *g* (male and female), respectively; *φ* is the dose–response coefficient between hair Hg levels and FHA risks; the subjective coefficient *ω* denotes the probability of the causality of the associations which reflects the substantial uncertainties resulting from limited epidemiological studies. The economic loss of the Hg-related health impacts (*H*) can be measured as follows:


(6) H=EL×ΔIQ+VSL×ΔCF,

where EL denotes the lifelong earning loss resulting from IQ decline which is assigned with a value of $18,832 (2008 value) per IQ 345 point (6). VSL denotes the value of statistical life, and a value of $6.3 million (2005 value) is adopted (19).

## Supplementary Material

pgad128_Supplementary_Data

## Data Availability

Access to all data required in this study is available in the Supplementary material and the research group website: https://www.ebmg.online/mercury; EDGAR global toxic pollutants emissions database: https://edgar.jrc.ec.europa.eu/; GTAP global MRIO database: https://www.gtap.agecon.purdue.edu/; Eora global supply chain database: https://worldmrio.com/; FAO/WHO global individual food consumption database: http://www.fao.org/nutrition/assessment/food-consumption-database/en/; World population prospects: https://population.un.org; and Fishbase database: https://www.fishbase.org. All model code is available at the research group website: https://www.ebmg.online/mercury.
