# Supplementary material for: International trade shapes global mercury–related health impacts
Source: PNAS Nexus. 2023 May 23;2(5):pgad128. doi: 10.1093/pnasnexus/pgad128 (PMC10205471; doi:10.1093/pnasnexus/pgad128)
Supplement: pgad128_Supplementary_Data [file pgad128_supplementary_data.pdf]

PNAS nexus

Supporting Information for

International trade shapes global mercury-related health impacts

Zhencheng Xing [1, †], Ruirong Chang [1, †], Zhengcheng Song [1], Yanxu Zhang [1, 2, *], Marilena Muntean [3], Kuishuang Feng [4], Yifan Liu [5], Zongwei Ma [5], Jigan Wang [6], Jie Zhang [6], Haikun Wang [1, 2, 7, *]

[1] Joint International Research Laboratory of Atmospheric and Earth System Sciences, School of Atmospheric Sciences, Nanjing University, Nanjing 210023, China
[2] Frontiers Science Center for Critical Earth Material Cycling, Nanjing University, Nanjing 210023, China
[3] European Commission, Joint Research Centre (JRC), Directorate for Energy, Transport and Climate, Air and Climate Unit, Via E. Fermi 2749, I-21027, Ispra, VA, Italy
[4] Department of Geographical Sciences, University of Maryland, College Park, MD 20742, USA
[5] State Key Laboratory of Pollution Control and Resource Reuse, School of the Environment, Nanjing University, Nanjing 210023, China
[6] School of Business, Hohai University, Nanjing 210023, China
[7] Collaborative Innovation Center of Climate Change, Jiangsu Province, Nanjing 210023, China
† These authors contributed equally to this work.
* Correspondence to: Yanxu Zhang and Haikun Wang.
Email: zhangyx@nju.edu.cn and wanghk@nju.edu.cn.

This PDF file includes:

Supporting text
Figures S1 to S11
Tables S1 to S4
SI References

Supporting Information Text

Extended illustration of uncertainty analysis

Our model results are subject to uncertainties from various sources, including the compilation of production-based emissions, the calculation of emissions embodied in trade, the simulation of the mercury transport model, the collection of food consumption data and food MeHg concentrations, and the evaluation of human health impacts and economic loss.

(1) The compilation of production-based Hg emission inventory is uncertain due to knowledge gaps regarding emission factor (e.g., Hg concentrations in fuel/raw materials), and activity rates. The uncertainties in both the activity data and emission factors used in the EDGARv4 mercury emission inventory are estimated using the default methodology recommended by IPCC (IPCC, 2006) with the lower and upper bounds of the 95% confidence interval from EMEP/EEA (EMEP/EEA, 2009) expressed as a percent relative to the mean for emission factors. The estimated uncertainty when combining the cement production, metal industry, combustion and waste incineration sectors ranges from −26 to +33% for OECD countries and Economies in Transition (Russia Federation, Ukraine and other eastern European countries) countries and −33 to +42% for Non-Annex I countries. Moreover, the uncertainty in the mercury emission inventory is expected to be much higher when considering the uncertainty in the activity data for artisanal and small scale gold production which is difficult to calculate due to the lack of official statistics in most countries (Muntean et al., 2014). However, updated activity data for sinter production in the iron & steel industry and new activity data for the ASGM sector from the Artisanal Gold Council (AGC, 2010) leads to an improvement in the uncertainty of EDGARv4.tox2 (Muntean et al., 2018). An uncertainty of [−33%, 42%] is used for the production-based emission inventory in this study.

(2) Estimates of emissions embodied in trade share the most uncertainties with production-based Hg emissions and contain an additional uncertainty from the MRIO model associated with inaccuracies in economic statistics, sectoral mapping, and data harmonization (Lenzen et al., 2010). Herwtich and Peters (2009) reported the coefficient of variation (CV) of trade coefficients have 10%-20% for GTAP-MRIO table. Based on the finding, Zhang et al. (2017) estimated uncertainty for consumption-based air pollution emissions for nations by adding a 13% of uncertainty to production-based emissions. A recent study reported uncertainty of Chinese province mercury footprint varying from 8% to 34% by using Monte Carlo simulation method (Zhang et al., 2019a). As the uncertainty for small emitters is larger than that of large emitters (Lenzen et al., 2010), the uncertainty for national mercury footprint in our study should be lower than 34%.

(3) Environmental Hg levels simulated by the comprehensive mercury transport model is affected by errors in emission inputs and the model representation of tropospheric chemical processes, especially Hg chemistry and physical processes such as vertical transport and wet scavenging. The modeled values in terms of surface total gaseous mercury, atmospheric wet deposition and marine surface MeHg concentration are comparable with the corresponding available observations (Figure S11), given a relatively large uncertainty range (Zhang et al., 2019b). Meanwhile, following the spirit of Chen et al. (2019) and Zhang et al. (2017), we apply the normalized root-mean-square deviation (NRMSD) between the simulations and observations over measurement sites to represent the uncertainties derived from the mercury transport model. The estimations are 29.1%, 62.4%, and 210.6% for surface total gaseous mercury, atmospheric wet deposition and marine surface MeHg concentration, respectively.

(4) The compilation of intake inventory of MeHg is subject to uncertainties in the MeHg concentrations of food products and intake rate of food products. MeHg concentrations of food products are collected from the literature. We use the variability of the log-transformed concentrations in each food category to represent its uncertainty (Zhang et al., 2021) and the estimation is [−37%, 63%]. We rely on the database of the United Nations' Food and Agriculture Organization (FAO) for food consumption. Compared with national data, the two data sources generally agree within a factor of 2. This reflects both the different survey methods and variability among the population (Cook et al., 2000; Grandjean, 2012). Based on the comparison between the food consumption data from UN FAO and national datasets (Zhang et al., 2021), the

difference between them is used to represent the uncertainty range of food consumption and the estimation is [−47%, 42%].

(5) The evaluation of human health impacts due to MeHg intake is subject to uncertainties in parameters used in the evaluation and the total deaths from fatal heart attacks collected from national statistics. We use the ranges (or standard deviations) of the dose-effect relationship between MeHg exposure and its health effect summarized by Chen et al. (2019) and Giang & Selin (2016), and the uncertainty estimation is [−59%, 147%]. For per-IQ earn loss, we use a high- and low-end value of $18,832 and $8013, respectively. The VSL per death ranges from $1 to $10 million following Giang & Selin (Chen et al., 2019). These economic valuation parameters lead to an uncertainty of [−70%, 26%] for economic loss.

Taken together these suggest greater uncertainty in estimates of the health impacts of specific scenarios. Ideally, we would have conducted a formal analysis of the propagation of uncertainty through our complete set of models (activity => emissions => concentrations => exposure => health impacts). Unfortunately, the computing time necessary to conduct such an analysis is prohibitive. Therefore, we consider the contribution of the data and parameters for food consumption, food MeHg concentrations, dose-effect relationship, and economic valuation to the total uncertainty. The overall uncertainty is estimated by a Monte Carlo approach. The health risk calculation is repeated for 1000 times with randomly sampled parameters for these four factors. The 2.5% and 97.5% percentiles of the calculated risk are taken as the overall uncertainty range (i.e., 95% confidence interval). The overall uncertainty is estimated at [−75.3%, 131.6%].

Limitations and prospects

In addition to the uncertainties discussed above, there are additional limitations in this study. Previous studies have shown that ecosystems can respond to changes in Hg inputs on timescales of years to decades (Vijayaraghavan et al., 2014; Selin et al., 2010). Hg concentrations in food products collected from the literature in this study were measured during recent decades and are used to represent the average condition of Hg risks in these decades. Hg emissions and environmental Hg pollution in 2011 can represent an average condition of Hg pollution during the last few decades. We establish relationships between Hg emissions and risks during the last few decades and do not consider the accurate lag time in the response between Hg emissions and risks for a specific time period. Additional measurements of yearly data on Hg concentrations in food products and accurate lag time in the response between Hg emissions and risks are needed in the future. Additionally, we assume that the food MeHg concentrations respond linearly to the level of Hg added to the ecosystem, which is an upper limit or a conservative estimation of the response relationship. Indeed, an ecosystem-level experimental study reveals a concaved curve in fish MeHg concentrations responding to the added Hg that is linearly increasing in a 15-year course (Blanchfield et al., 2022). The assumption can be improved when more data are available in the future. We consider no time lags among environmental levels, food concentrations, and human exposure, which might be acceptable as most of the food items (e.g., rice, aqua- and mariculture) are harvested and consumed within a few years. Moreover, the ecosystem-level study also shows a timely response of fish MeHg to the addition of Hg to the environment (Giang & Selin, 2016).

This study develops a more comprehensive assessment method to investigate the chain of the biogeochemical Hg cycle from economic activities to human health at the global level. In addition to economic supply chains and Hg emission sources, the biogeochemical Hg cycle and related adverse health impacts (especially the human exposures) are also influenced by multiple extrinsic and intrinsic factors. The extrinsic factors include climate change, land use change, hydrologic management, invasive species, and food consumption & dietary habits. The intrinsic factors include genetics, gastrointestinal assimilation, microbiome, nutrients & co-exposures to other contaminants, and co-exposures to other diseases (Grandjean, 2012). However, this study aims to identify the role of international trade in relocation of global Hg emissions, pollution, exposure, and related health burden. Therefore, these extrinsic and intrinsic factors are assumed to be consistent under the “with trade” and “no trade” scenarios, and have not been taken into consideration in this study. Moreover, the mechanisms of some intrinsic factors (e.g., microbiome) still remain unknown (Eagles-Smith et al., 2018; Madan et al., 2012), which prevents

the adoption of these intrinsic factors in the assessment of Hg-related health risks, especially for studies on large human communities at the macro scale (Grandjean, 2012). Nevertheless, the values of parameters describing certain extrinsic factors can be incorporated into the model to track the changes in Hg cycle and related health risks in the context of rapid global changes. Meanwhile, the intrinsic factors can also be practicably incorporated into the model, when future studies can clearly characterize their dynamics, mechanisms, and modelling methods.

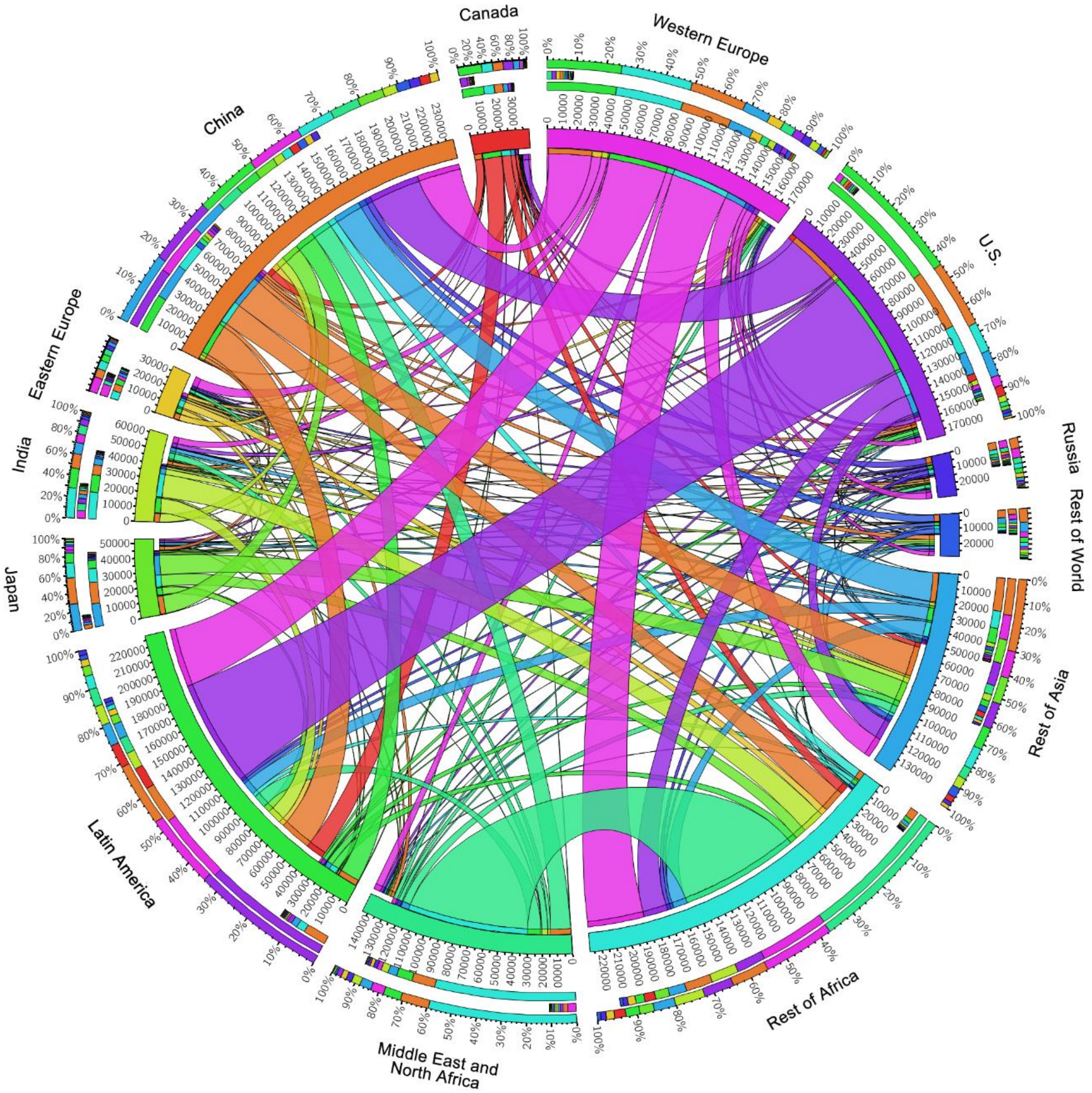

Figure S1. Bilateral flows of Hg emissions embodied in the international trade between 13 world regions. The arc length of each region denotes Hg emissions embodied in its exports and imports. The chord linking two regions provides the information for their embodied Hg flows to each other. The width of links denotes the magnitude of Hg transfer flows. In the clockwise direction of the diagram, the links show outflows first, followed by inflows. Hg transfer from a source region is indicated with a link emanating from the arc of the same color. A recipient region is indicated with an arc from the link of a different color.

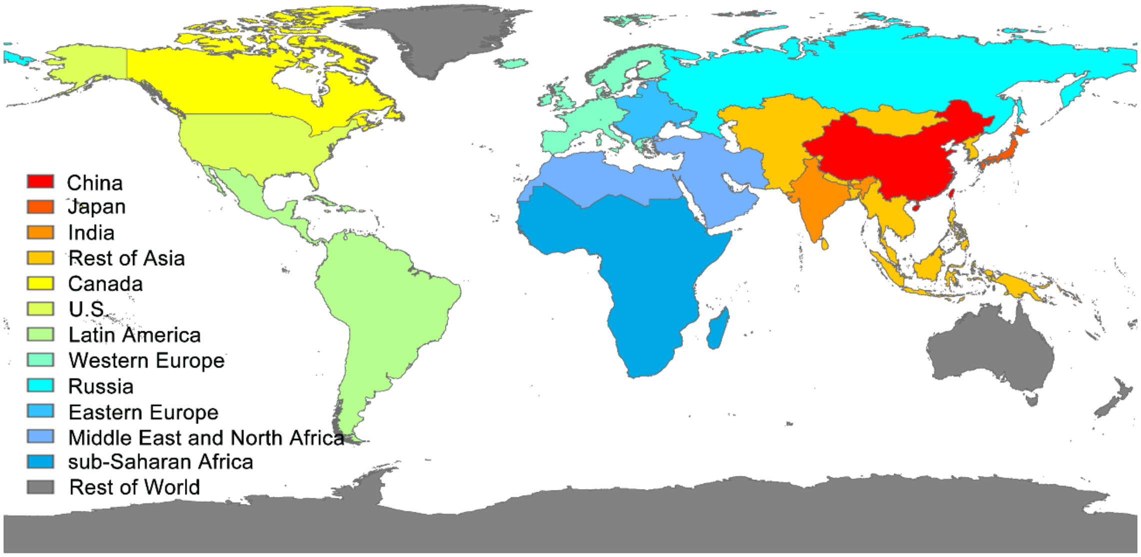

Figure S2. Definition of the 13 world regions used here.

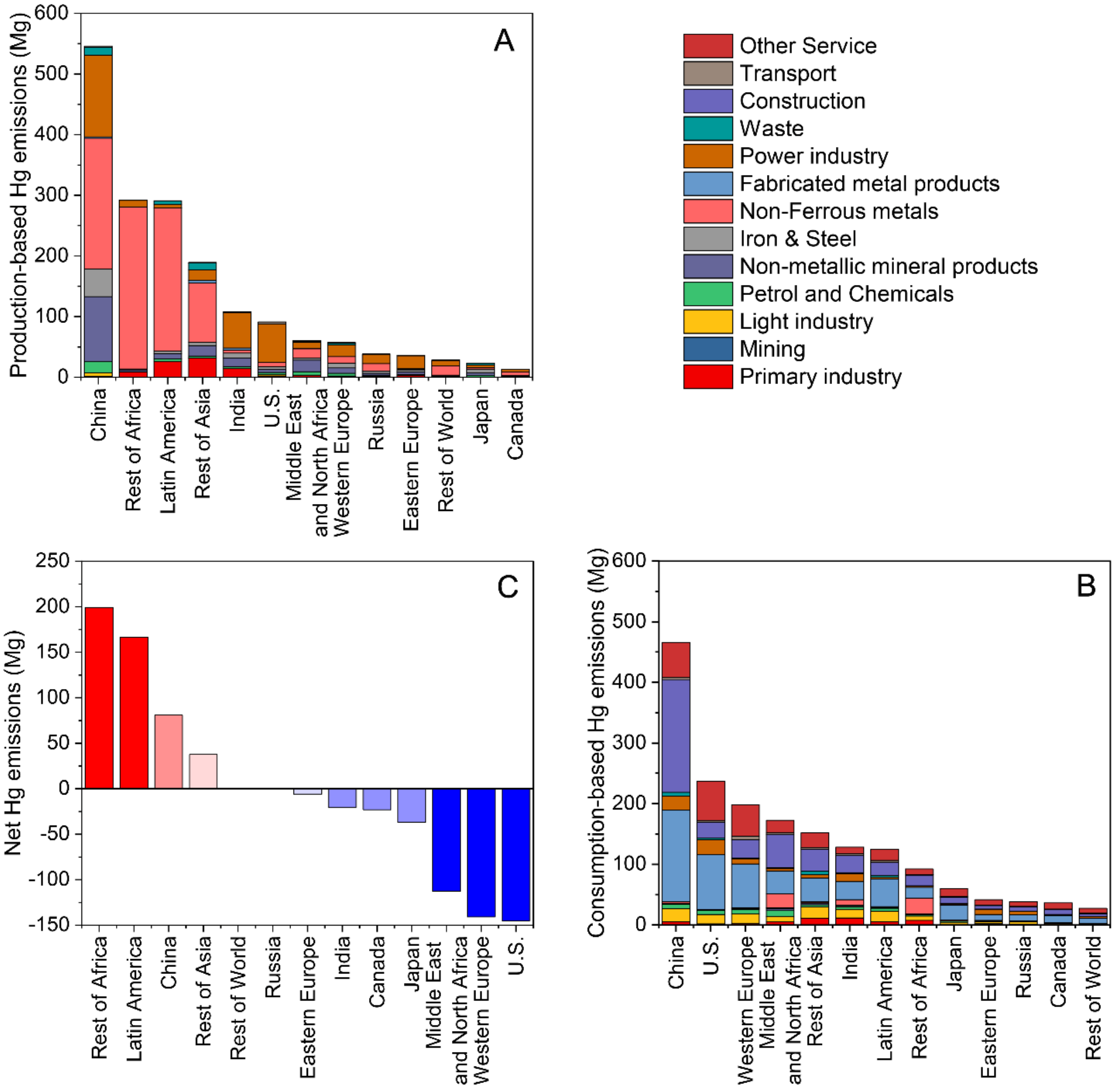

Figure S3. Comparison of production-based and consumption-based emissions for the 13 world regions. (A) Production-based Hg emissions of 13 sectors in 13 world regions. (B) Consumption-based Hg emissions of 13 sectors in 13 world regions. (C) Net Hg emissions (the difference between production-based and consumption-based Hg emissions) in 13 world regions.

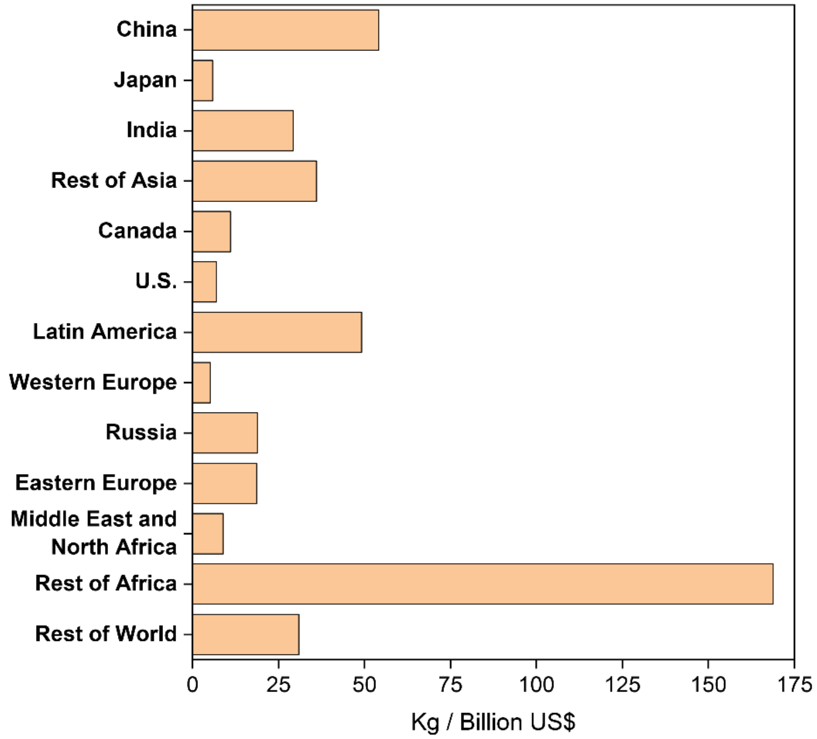

Figure S4. Hg emission intensity of the 13 world regions.

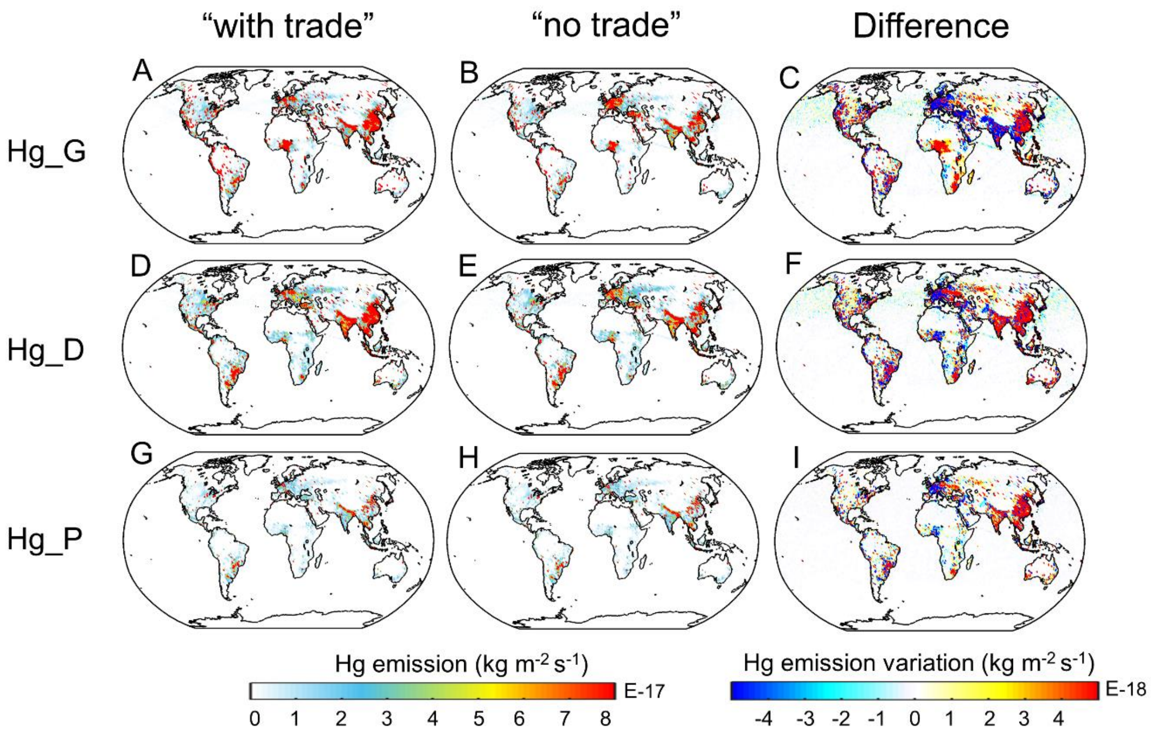

Figure S5. Spatial distribution of Hg emission inventory under "with trade" and "no trade" scenarios and the differences therebetween. The rows (A–C), (D–F), and (G–I) are for gaseous elemental, gaseous oxidized, and particle-bound Hg, respectively. For each row, the three panels denote the spatial distribution of Hg emissions for "with trade" and "no trade" scenarios and the differences therebetween.

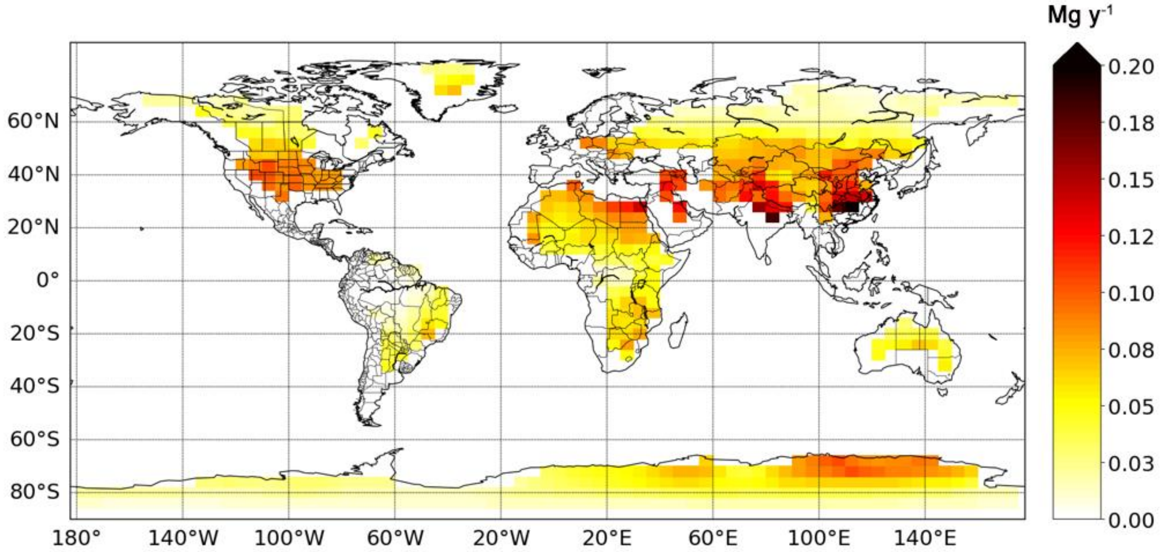

Figure S6. The spatial distribution of atmospheric Hg oxidation rates in the surface atmosphere.

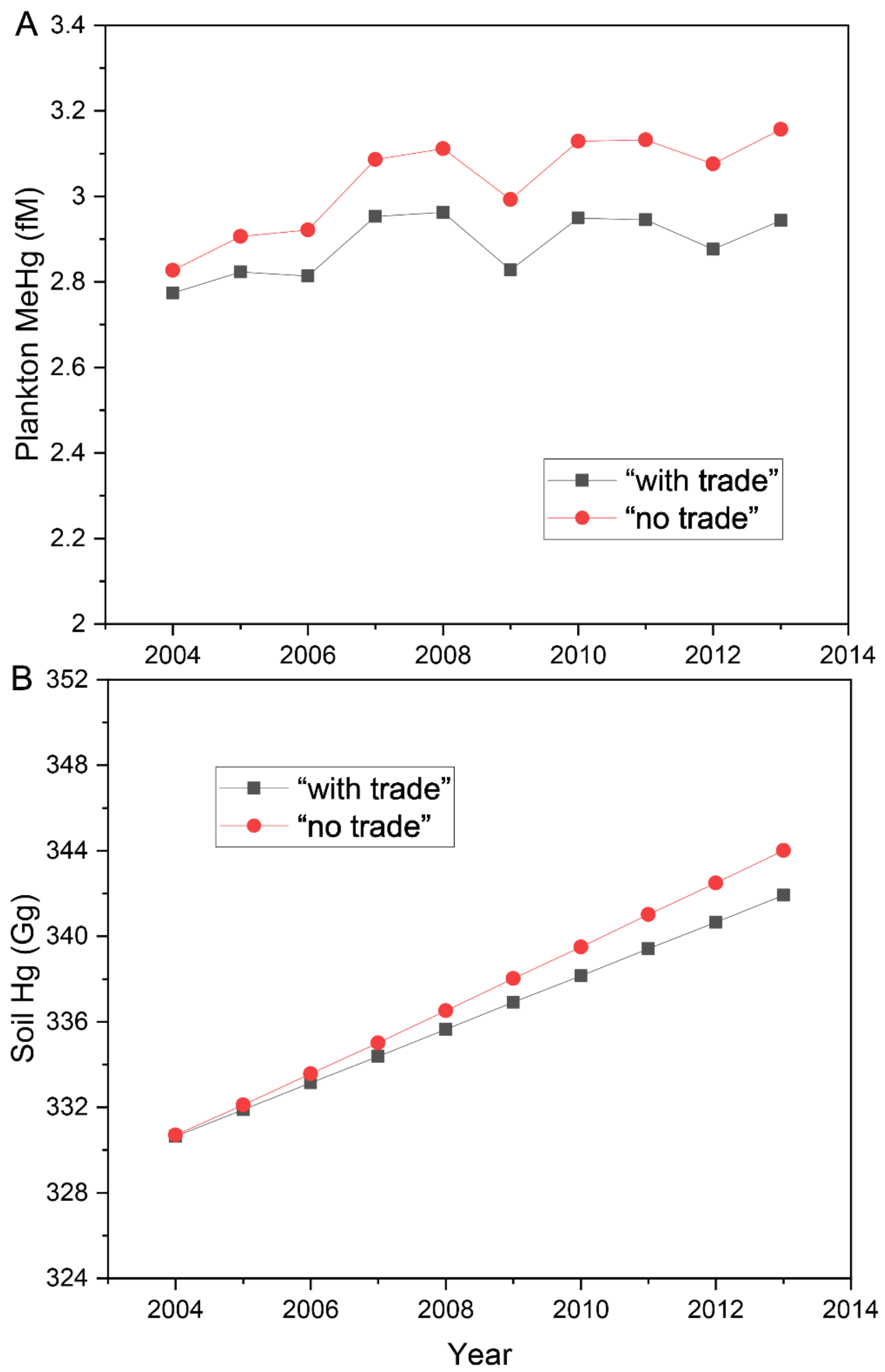

Figure S7. The global planktonic MeHg (A) and soil Hg (B) levels under "with trade" and "no trade" scenarios.

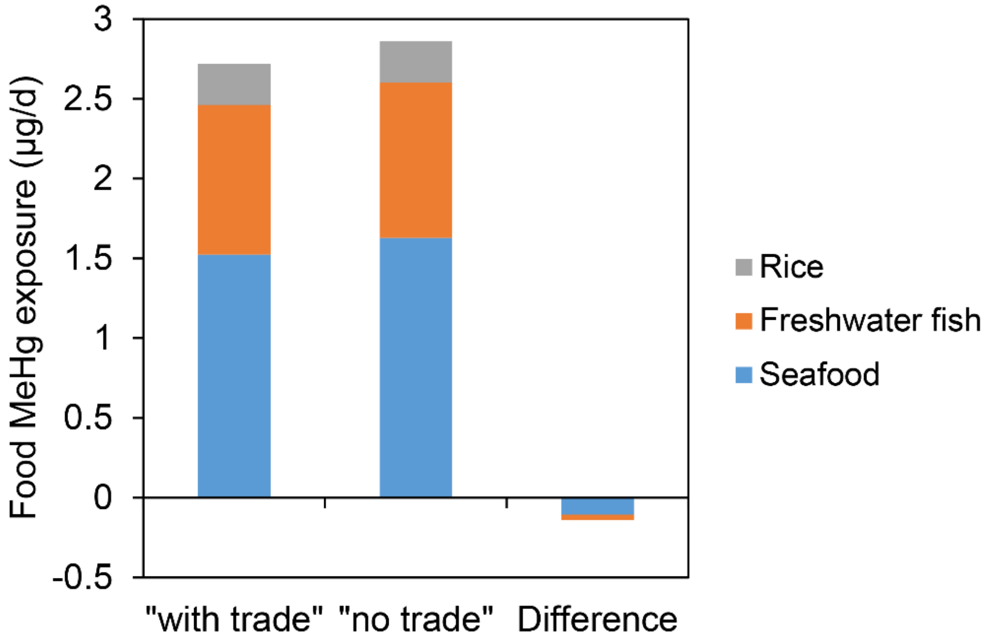

Figure S8. Per capita food MeHg exposure from seafood, freshwater fish and rice for "with trade" and "no trade" scenarios and the differences therebetween at the global level.

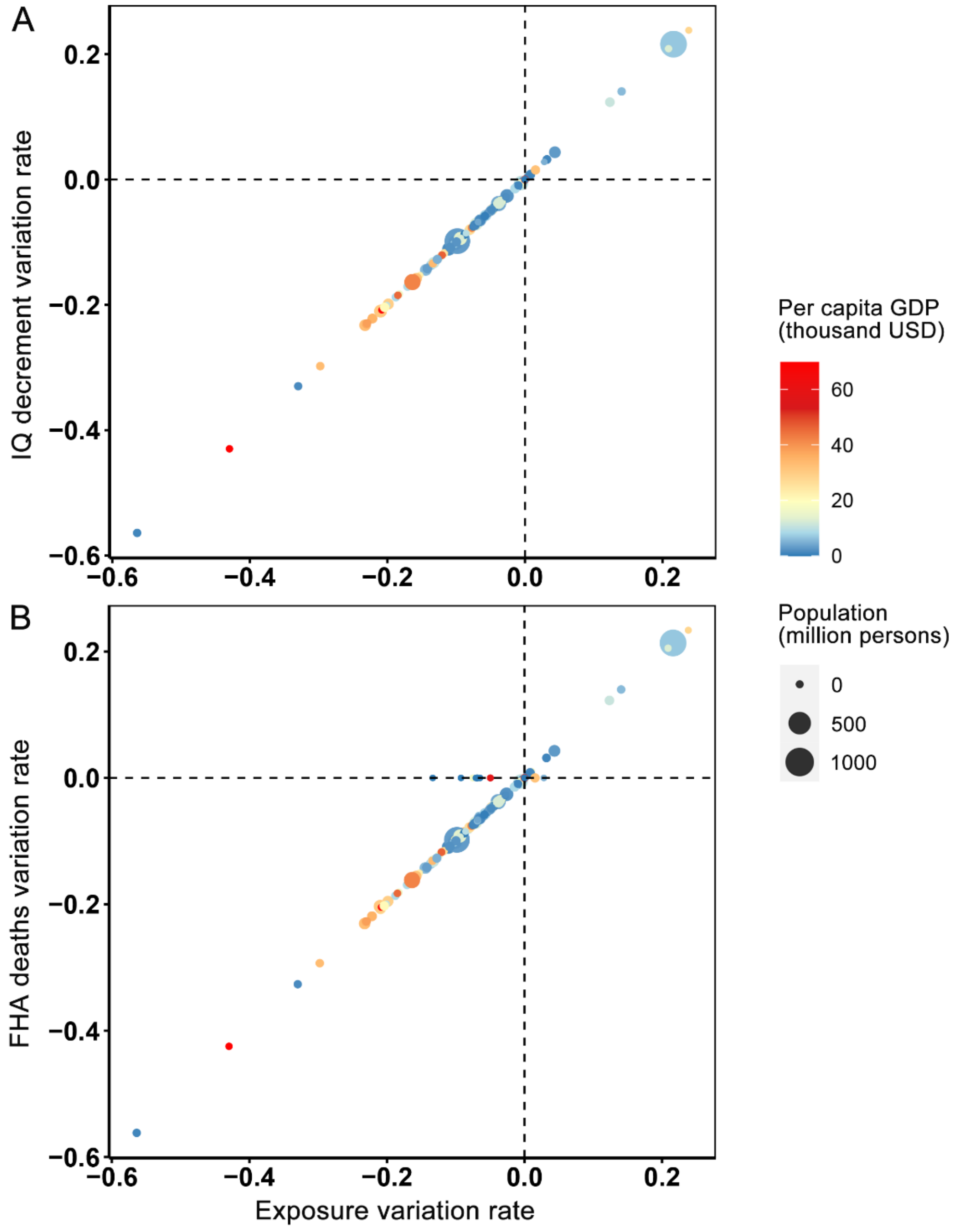

Figure S9. Contrasting changes in MeHg exposure and health impact across the world's countries. The figure shows the relative changes in regional MeHg exposure and (A) IQ decrement or (B) FHA deaths resulting from international trade for each country. The color of the dots denotes the magnitude of per capita GDP, and the size of the dots denotes the amount of population.

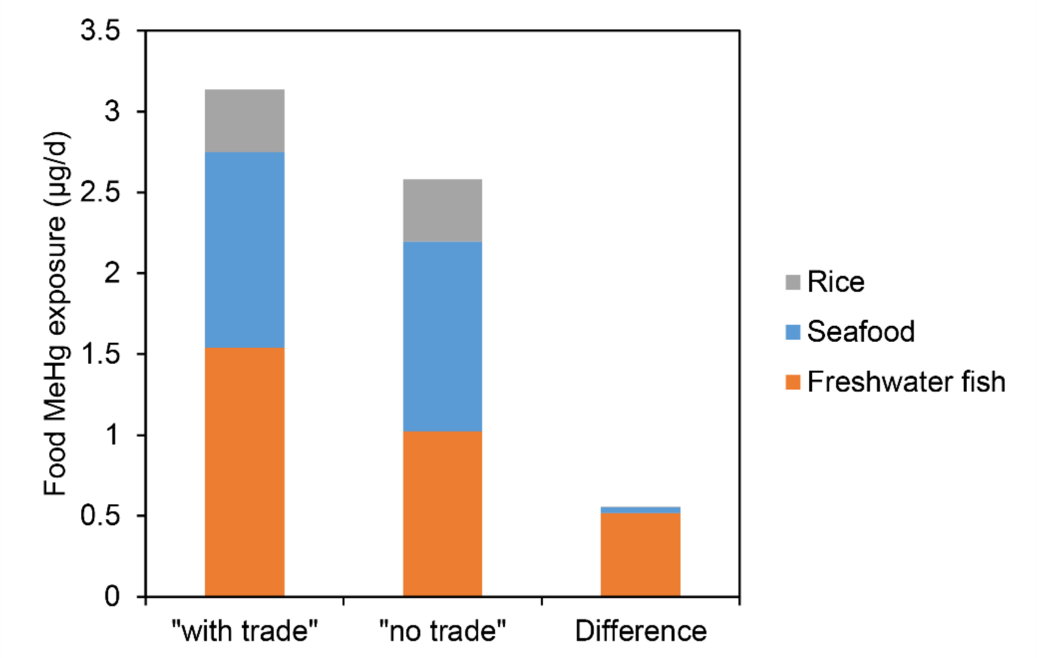

Figure S10. Hg emission intensity of the 13 world regions.

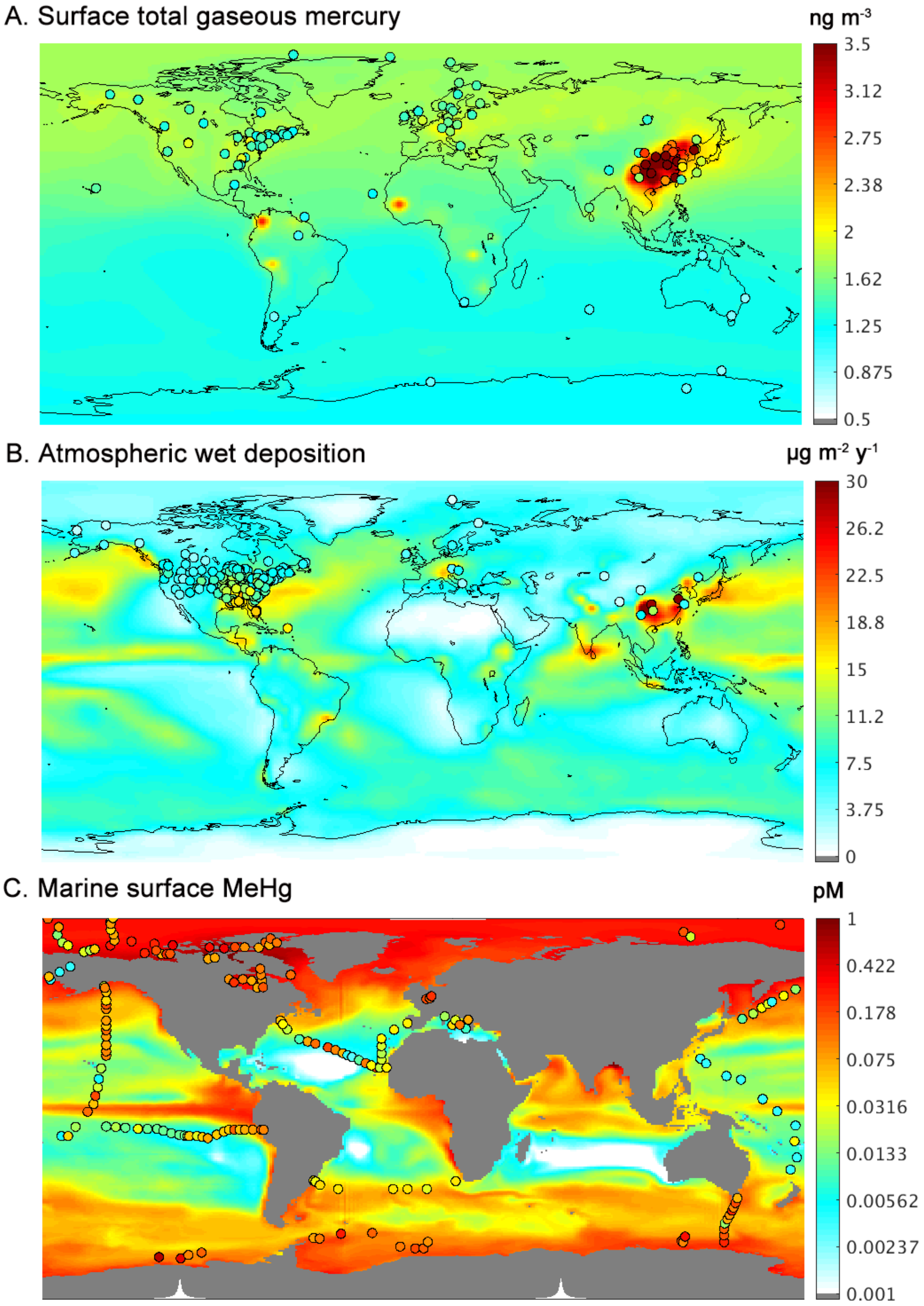

Figure S11. Comparison against observed (A) surface total gaseous mercury, (B) wet deposition and (C) seawater MeHg concentrations in the surface ocean (top 100 m, pM = pico Mol/L). The data sources are summarized by our previous studies (Zhang & Zhang, 2022; Zhang et al., 2020).

Table S1. Description of “with trade” and “no trade” scenarios.

Scenarios	Description	Emissions
“with trade”	The actual scenario with the existence of international trade	Regional emissions accounted by production
“no trade”	The counterfactual scenario with an absence of international trade	Regional emissions accounted by consumption

Note: The global total emission quantity under “with trade” and “no trade” scenarios are equal.

Table S2. Sectors of Hg emissions in EDGAR.

EDGAR sector	IPCC code	Description
AWB	4F	Agricultural waste burning
CHE	2B5g	Production of chemicals
ENE	1A1a	Energy industry: power generation
IND	1A2	Combustion in industry
IRO	2C1	Production of iron and steel
NFE	2C5	Production of non-ferrous metals
NMM	2A	Production of non-metallic minerals
RCO	1A4	Small combustion: buildings
REF	1A1b	Refineries
SWD	6C	Waste
TNR	1A3d	Non-road transport
TNR	1C2	International shipping (see SEA)
TRF	1A1c, 1A5, 1B1	Transformation industry
TRO	1A3b	Road transport

Table S3. Sector mapping.

Code	Aggregated sectors	GTAP sectors
1	Agriculture	Rice: seed, paddy (not husked); Wheat: seed, other; Other Grains: maize (corn), sorghum, barley, rye, oats, millets, other cereals; Veg & Fruit: vegetables, fruit and nuts, edible roots and tubers, pulses; Oil Seeds: oil seeds and oleaginous fruit; Cane & Beet: sugar crops; Fibres crops; Other Crops
2	Production of Cattle	Cattle: bovine animals, live, other ruminants, horses and other equines, bovine semen
3	Production of Other Animal Products	Other Animal Products
4	Production of Raw milk	Raw milk
5	Production of Wool	Wool: wool, silk, and other raw animal materials used in textile
6	Forestry	Forestry: forestry, logging and related service activities
7	Fishing	Fishing: hunting, trapping and game propagation including related service activities, fishing, fish farms; service activities incidental to fishing
8	Coal mining	Coal: mining and agglomeration of hard coal, lignite and peat
9	Oil extraction	Oil: extraction of crude petroleum, service activities incidental to oil and gas extraction excluding surveying (part)
10	Gas extraction	Gas: extraction of natural gas, service activities incidental to oil and gas extraction excluding surveying (part)
11	Other Mining Extraction	Other Mining Extraction (formerly omn): mining of metal ores; other mining and quarrying
12	Processing of Food products	Cattle Meat; Other Meat; Vegetable Oils; Milk: dairy products; Processed Rice: semi- or wholly milled, or husked; Sugar and molasses; Other Food
13	Production of Beverages and Tobacco products	Beverages and Tobacco products
14	Manufacture of textiles	Manufacture of textiles
15	Manufacture of wearing apparel	Manufacture of wearing apparel
16	Manufacture of leather and related products	Manufacture of leather and related products
17	Manufacture of wood and related products	Lumber: manufacture of wood and of products of wood and cork, except furniture; manufacture of articles of straw and plaiting materials
18	Production of paper and printing	Paper & Paper Products: includes printing and reproduction of recorded media
19	Manufacture of coke and refined petroleum products	Petroleum & Coke: manufacture of coke and refined petroleum products
20	Manufacture of chemicals and chemical products	Manufacture of chemicals and chemical products; Manufacture of pharmaceuticals, medicinal chemical and botanical products; Manufacture of rubber and plastics products
21	Manufacture of other non-metallic mineral products	Manufacture of other non-metallic mineral products
22	Production and casting of Iron and Steel	Iron & Steel: basic production and casting

23	Production and casting of copper, aluminium, zinc, lead, gold, and silver	Non-Ferrous Metals: production and casting of copper, aluminium, zinc, lead, gold, and silver
24	Manufacture of metal products	Manufacture of fabricated metal products, except machinery and equipment
25	Manufacture of machinery and equipment	Manufacture of computer, electronic and optical products; Manufacture of electrical equipment; Manufacture of machinery and equipment n.e.c.
26	Manufacture of transport equipment	Manufacture of motor vehicles, trailers and semi-trailers; Manufacture of other transport equipment
27	Other Manufacturing	Other Manufacturing: includes furniture
28	Production and supply of electricity, gas and steam	Electricity; steam and air conditioning supply; Gas manufacture, distribution
29	Water supply; sewerage, waste management and remediation activities	Water supply; sewerage, waste management and remediation activities
30	Construction	Construction: building houses factories offices and roads
31	Wholesale and retail trade; repair of motor vehicles and motorcycles	Wholesale and retail trade; repair of motor vehicles and motorcycles
32	Accommodation, Food and service activities	Accommodation, Food and service activities
33	Land transport and transport via pipelines	Land transport and transport via pipelines
34	Non-road transport	Water transport; Air transport
35	Warehousing, post and telecommunications	Warehousing and support activities; Information and communication
36	Finance and insurance	Other Financial Intermediation: includes auxiliary activities but not insurance and pension funding; Insurance (formerly isr): includes pension funding, except compulsory social security
37	Real estate activities	Real estate activities
38	Other Business Services nec	Other Business Services nec
39	Other service activities	Recreation & Other Services; Other Services (Government); Education; Human health and social work; Dwellings: ownership of dwellings (imputed rents of houses occupied by owners)

Table S4. Correspondence relationships between EDGAR emission sources and sectors of the MRIO table.

EDGAR Emission sources	Corresponding sector in the MRIO table
Agricultural waste burning	Agriculture
Production of chemicals	Manufacture of chemicals and chemical products
Energy industry: power generation	Production and supply of electricity, gas and steam
Combustion in industry: manufacturing industries and construction	Disaggregated to 18 manufacturing industries and construction
Production of iron and steel	Production and casting of Iron and Steel
Production of non-ferrous metals	Production and casting of copper, aluminum, zinc, lead, gold, and silver
Production of non-metallic minerals	Manufacture of other non-metallic mineral products
Refineries	Manufacture of coke and refined petroleum products
Waste	Water supply; sewerage, waste management and remediation activities
Non-road transport	Non-road transport
Transformation industry	Manufacture of coke and refined petroleum products
Road transport	Land transport and transport via pipelines

Note: The emissions from “Small combustion: buildings” and “International shipping (see SEA)” are assumed to remain unchanged under production- and consumption-based accounting, and therefore not incorporated into the MRIO model. The emissions from “Combustion in industry” are disaggregated to 19 related industries in each country based on the Hg emission inventory obtained from Eora which provides sector-level Hg emissions from energy combustion for each country.

SI References

AGC, 2010. Global Database on Mercury Emissions from Artisanal and Small Scale Mining (ASGM). Artisanal Gold Council info retrived from: www.mercurywatch.org [on 15 October 2013].

Blanchfield, P. J., Rudd, J. W., Hrenchuk, L. E., Amyot, M., Babiarz, C. L., Beaty, K. G., ... & Tate, M. T. (2022). Experimental evidence for recovery of mercury-contaminated fish populations. Nature, 601(7891), 74-78.

Chen, L., Liang, S., Liu, M., Yi, Y., Mi, Z., Zhang, Y., ... & Yang, Z. (2019). Trans-provincial health impacts of atmospheric mercury emissions in China. Nature communications, 10(1), 1-12.

Cook, A., Pryer, J., & Shetty, P. (2000). The problem of accuracy in dietary surveys. Analysis of the over 65 UK National Diet and Nutrition Survey. Journal of Epidemiology & Community Health, 54(8), 611-616.

Eagles-Smith, C. A., Silbergeld, E. K., Basu, N., Bustamante, P., Diaz-Barriga, F., Hopkins, W. A., ... & Nyland, J. F. (2018). Modulators of mercury risk to wildlife and humans in the context of rapid global change. Ambio, 47(2), 170-197.

EMEP/EEA. EMEP/EEA CORINAIR emission inventory guidebook. Technical report No 9/ 2009. European Environment Agency; 2009 [http://www.eea.europa.eu/publications/emep-eea-emission-inventory-guidebook-2009].

Giang, A., & Selin, N. E. (2016). Benefits of mercury controls for the United States. Proceedings of the National Academy of Sciences, 113(2), 286-291.

Grandjean, A. C. (2012). Dietary intake data collection: challenges and limitations. Nutrition reviews, 70(suppl_2), S101-S104.

Hertwich, E. G., & Peters, G. P. (2009). Carbon footprint of nations: a global, trade-linked analysis. Environmental science & technology, 43(16), 6414-6420.

IPCC. IPCC guidelines for national greenhouse gas inventories. IPCC; 2006 [http://www. ipcc-nggip.iges.or.jp/public/2006gl/index.html].

Lenzen, M., Wood, R., & Wiedmann, T. (2010). Uncertainty analysis for multi-region input–output models–a case study of the UK's carbon footprint. Economic Systems Research, 22(1), 43-63.

Madan, J. C., Farzan, S. F., Hibberd, P. L., & Karagas, M. R. (2012). Normal neonatal microbiome variation in relation to environmental factors, infection and allergy. Current opinion in pediatrics, 24(6), 753.

Muntean, M., Janssens-Maenhout, G., Song, S., Giang, A., Selin, N. E., Zhong, H., ... & Dentener, F. (2018). Evaluating EDGARv4. tox2 speciated mercury emissions ex-post scenarios and their impacts on modelled global and regional wet deposition patterns. Atmospheric Environment, 184, 56-68.

Muntean, M., Janssens-Maenhout, G., Song, S., Selin, N. E., Olivier, J. G., Guizzardi, D., ... & Dentener, F. (2014). Trend analysis from 1970 to 2008 and model evaluation of EDGARv4 global gridded anthropogenic mercury emissions. Science of the Total Environment, 494, 337-350.

Selin, N. E., Sunderland, E. M., Knightes, C. D., & Mason, R. P. (2010). Sources of mercury exposure for US seafood consumers: implications for policy. Environmental health perspectives, 118(1), 137-143.

Vijayaraghavan, K., Levin, L., Parker, L., Yarwood, G., & Streets, D. (2014). Response of fish tissue mercury in a freshwater lake to local, regional, and global changes in mercury emissions. Environmental toxicology and chemistry, 33(6), 1238-1247.

Zhang, H., He, K., Wang, X., & Hertwich, E. G. (2019a). Tracing the uncertain Chinese mercury footprint within the global supply chain using a stochastic, nested input–output model. Environmental science & technology, 53(12), 6814-6823.

Zhang, P., & Zhang, Y. (2022). Earth system modeling of mercury using CESM2–Part 1: Atmospheric model CAM6-Chem/Hg v1. 0. Geoscientific Model Development, 15(9), 3587-3601.

Zhang, Q., Jiang, X., Tong, D., Davis, S. J., Zhao, H., Geng, G., ... & Guan, D. (2017). Transboundary health impacts of transported global air pollution and international trade. *Nature*, *543*(7647), 705-709.

Zhang, Y., Horowitz, H., Wang, J., Xie, Z., Kuss, J., & Soerensen, A. L. (2019b). A coupled global atmosphere-ocean model for air-sea exchange of mercury: insights into wet deposition and atmospheric redox chemistry. Environmental science & technology, 53(9), 5052-5061.
Zhang, Y., Soerensen, A. L., Schartup, A. T., & Sunderland, E. M. (2020). A global model for methylmercury formation and uptake at the base of marine food webs. Global Biogeochemical Cycles, 34(2), e2019GB006348.
Zhang, Y., Song, Z., Huang, S., Zhang, P., Peng, Y., Wu, P., ... & Li, P. (2021). Global health effects of future atmospheric mercury emissions. Nature communications, 12(1), 1-10.
